# Mesoscopic elasticity controls dynamin-driven fission of lipid tubules

**DOI:** 10.1038/s41598-024-64685-2

**Published:** 2024-06-18

**Authors:** Marco Bussoletti, Mirko Gallo, Matteo Bottacchiari, Dario Abbondanza, Carlo Massimo Casciola

**Affiliations:** 1 Department of Mechanical and Aerospace Engineering, Sapienza University of Rome, Rome, Italy; 2 Department of Basic and Applied Sciences for Engineering, Sapienza University of Rome, Rome, Italy

**Keywords:** Canham Helfrich, Phase field, Fluid lipid bilayer, Membrane fission, Topological transition, Biological physics, Applied mathematics, Computational biophysics, Membrane biophysics

## Abstract

Mesoscale physics bridges the gap between the microscopic degrees of freedom of a system and its large-scale continuous behavior and highlights the role of a few key quantities in complex and multiscale phenomena, like dynamin-driven fission of lipid membranes. The dynamin protein wraps the neck formed during clathrin-mediated endocytosis, for instance, and constricts it until severing occurs. Although ubiquitous and fundamental for life, the cooperation between the GTP-consuming conformational changes within the protein and the full-scale response of the underlying lipid substrate is yet to be unraveled. In this work, we build an effective mesoscopic model from constriction to fission of lipid tubules based on continuum membrane elasticity and implicitly accounting for ratchet-like power strokes of dynamins. Localization of the fission event, the overall geometry, and the energy expenditure we predict comply with the major experimental findings. This bolsters the idea that a continuous picture emerges soon enough to relate dynamin polymerization length and membrane rigidity and tension with the optimal pathway to fission. We therefore suggest that dynamins found in in vivo processes may optimize their structure accordingly. Ultimately, we shed light on real-time conductance measurements available in literature and predict the fission time dependency on elastic parameters.

## Introduction

Discovered in 1989, dynamin is the first protein shown to catalyze fission of lipid vesicles^1^. Its ability to promote tubulation by helically polymerizing on compliant, high curvature, and cylindrical lipid substrates^2^ and to drive their constriction and fission via a series of conformational changes upon GTP (guanosine triphosphate) hydrolysis^3–7^ grants the dynamin a key role in the fascinating choreography of endocytosis. Indeed, dynamin is recruited on and helically wraps around the neck of clathrin-^8^ or caveolin-induced^9^ buds, for instance, eventually finalizing the detachment of endocytic vesicles^3,10,11^ necessary for nutrient uptake, synaptic transmission, and intracellular trafficking^12–14^. Altogether, these aspects link mutations or misregulated expressions of dynamin to a plethora of neurodegenerative disorders^15^ and myopathies^16^.

Despite its physiological relevance and ubiquity, a thorough picture of how dynamin drives constriction and cleavage of membrane necks is still missing, especially for what concerns the comprehension and quantification of energy barriers, forces, minimal machinery, and role of geometrical aspects^11,14^. As a matter of fact, whether it is on necks developed in the final stages of clathrin-mediated endocytosis^12^ or their in vitro surrogates, i.e. lipid tubules with radii in the range of 10 to 30 nm ^2^, the dynamin monomers polymerize into helices spanning from tens to hundreds of nanometers along the tubule’s axis. Therefore, the GTP-dependent molecular activity of dynamin meets the lipids’ response on a mesoscopic scale where elastic features start playing a role. In this concern, continuum elasticity interests a nourished cohort of theoretical and experimental studies dealing with both membrane- and polymer-related mechanics^17–26^. Such coarse-grained descriptions not only possess the ability to effectively portray the behavior of the system at this scale but also operate on a reduced amount of variables and complexity^27,28^. Pursuing this motif, we aim to recognize a common mechanism underlying dynamin-driven fission and emerging from a scale where the granular nature of matter fades into a more diffuse and continuous one. As such, it is pivotal to characterize the influence of the key mesoscale quantities defining the system, such as dynamin’s polymerization length (or number of rungs), tubule radius as well as membrane rigidity and tension. Each one of these variables eludes the reach of atomistic approaches when dealing with full-scale processes and rightfully enters the grasp of continuum elasticity, which is thence expected to bridge the gap toward experimentally accessible observables.

Indeed, experimental techniques may either allow to spatially reconstruct the dynamin-lipid assembly down to a subnanometer detail employing, most often, mutated compounds at the price of a practically unfeasible observation of fast, dynamic processes^4,7,11,18,21,24,25,29–35^ or probe the system evolution with sufficiently narrow time resolutions while being limited to mesoscale or integral observables, e.g., tubule’s radius and integrity and dynamin’s length^6,12,18,19,36–39^. Such limits may be transcended by full atomistic molecular dynamics techniques, which are invaluable tools for shedding light on the biochemistry of protein–lipid interactions and determining the nature of the GTP-activated conformational changes^24,29,31^. Recent advancements, amidst the other possibilities^19,37,40–43^, substantiate the hypothesis of a ratchet-like power stroke taking place between the cross-linked dimers of adjacent rungs composing the helix^4,18,24,32,39^. Furthermore, through a combination of experimental and numerical investigations^24^, these GTPase power strokes were shown to be intense enough to actively constrict the membrane. Still, the full framework encompassing both constriction and fission of the lipid tubular structure remains unclear. Nonetheless, the computational cost associated to atomistic techniques imposes limitations on the accessible spatiotemporal scales and, in order to deal with the full-scale evolution of the tubule severing, rather strong coarse-graining approaches shall be deployed^25,37,40,44,45^.

**Figure 1:**
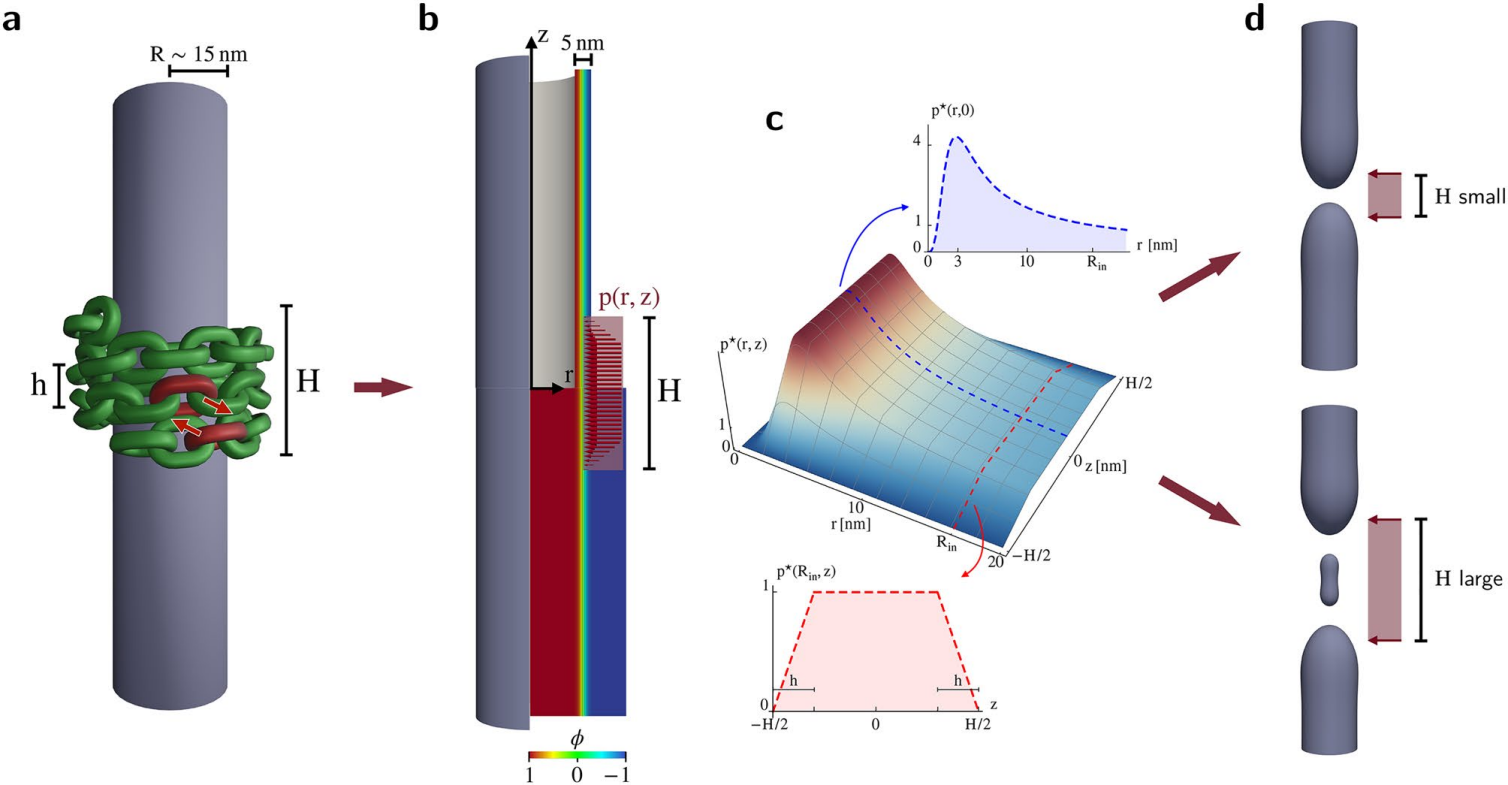
Outline of the mesoscopic elastic picture. (**a**) The mesoscale interpretation of dynamin as a chain-like helix structure where molecular details are replaced by its essential geometric characteristics, like internal and external radii, pitch *h*, polymerization length *H*, and number of dimers per turn $N_d$. The forces exchanged by dimers of adjacent rungs following GTP hydrolysis are highlighted in red for a pair of chain links^24^. This same interaction is repeated for each pair of dimers, resulting in a statistically zero net force for dimers in the inner turns. (**b**) At the mesoscale, the lipid bilayer behaves as a continuum elastic medium. Based on the celebrated Canham^48^ and Helfrich^49^ elasticity model, the membrane mechanics is here portrayed through a diffuse interface approach capable of following the full-scale processes of constriction and topological rearrangement in a regularized way^50^. This $5\,\mathrm {nm}$-thick diffuse interface, highlighted in the upper half of the contour plot, is defined by the order parameter $\phi (\varvec{x})$, whose complete structure is shown in the lower half of the contour plot ranging from $-1$ in the blue, external region to $+\,1$ in the red, internal one. (**c**) Focusing on a coarser and more diffuse picture of the dynamin action onto lipid tubules, it is possible to retrieve a pressure, *p*(*r*, *z*), that depends on the position along the tubule axis, as shown in the lower red graph, and its local radius, shown in the upper blue graph (see Supplementary Information for additional details). The intensity of this pressure depends on the above-mentioned key quantities as well as on the tangential component of the force originating from the ratchet-like power strokes, $F_{\tau }\approx 2.5\,\mathrm {pN}$^24^, and is therefore normalized as $p(r,z)=p^{\star }(r,z)\times p(R_\text{in},0)$, where $p(R_\text{in},0)=N_d F_{\tau }/(h R_\text{in})$ is its value at the center of the initial tubule with radius $R_\text{in}$. (**d**) The outcomes of the constriction phase could result in the severing of the lipid tubule. As will be shown later in the article, different fission pathways may delineate depending on mesoscopic quantities such as the polymerization length.

Within this work, by sacrificing a sizeable level of detail in the protein machinery, we build a mesoscopic model based on a consistent elasticity description and able to retain the key features of dynamin-induced constriction. In particular, we develop a protein-interaction term to be coupled with a diffuse interface, Ginzburg–Landau type of free energy which reproduces the results of classical elasticity membrane models^46,47^ —namely the Canham–Helfrich Hamiltonian^48,49^— and overcomes the associated limitations by accounting for topology-related effects across membrane severing^50^. Analogous approaches were recently proved effective in thoroughly reproducing mesoscale phenomena like, e.g., boiling^51^, water cavitation^52,53^, vapor bubble nucleation^54,55^, Rayleigh-Plateau instability^56^, crystal growth^57^, and dielectrics breakdown^58^. Figure 1 depicts the outline and general idea of this article. For the purpose of aiding a mechanical comprehension of the continuous model construction, the dynamin complexity is first shrunk down to a simple chain-like picture where the ratchet power strokes take place between chain links of adjacent turns (a pair of which is highlighted in red in Fig. 1a). A further coarse-graining step loses focus on the discrete nature of the chain and enforces a diffuse paradigm portraying the effect of conformational changes onto the membrane (Fig. 1, panels b,c), eventually resulting in the constriction and fission of the tubule (Fig. 1d).

We investigate whether pure constriction may play as the main mechanism leading to fission and what are the implications of different polymerization lengths on effectiveness and efficiency. Experimentally available results on phenomenology, critical neck radii, and energy expenditure estimates are shown to corroborate the picture here proposed. Unexpectedly, some insights on the location of fission nucleation sites are retrieved, shedding light on the role of membrane tension as well as nonlinear elasticity effects in determining whether severing is expected to occur at the middle or at the edge of dynamin coats, a duality first recognized by Pannuzzo et al.^40^. All these aspects converge in reproducing the experimentally measured time required for completing fission, eventually evidencing its relationship with the key mechanical properties of the system.

## Results

### The mesoscopic elastic picture

Five domains constitute the prototypical dynamin monomer^11,29–31^. Among these, the GTPase (G) domain is responsible for the power-stroke generation, then transmitted to the underlying lipid substrate by the membrane-binding pleckstrin homology (PH) domain^7^. The detail of the model construction, starting from the mechanical role of each domain, may be found in the Supplementary Information. For our purposes, however, it is worth knowing that, as revealed by cryo-electron microscopy, dynamin monomers crisscross associate in an anti-parallel dimer, itself constituting the building block of the helix^7,14,31^, and thus expose the power units (the G domains) to the adjacent rungs. From a mechanical point of view, these molecular motors connect the adjacent rungs and apply a shearing force on them, thus inducing a longitudinal tension. This is balanced by a pressure acting on the membrane. The tension builds up in the two extremal rungs and is constant in the inner ones, hence self-equilibrated in the longitudinal direction.

In other words, since the chain sustains internal tension but exhibits no significant bending reactions^24,29^, one can retrieve the pressure experienced by the lipid bilayer for polymers completing at least one full turn. It should be noticed that the shear stress on the membrane may affect the fission process as well, as suggested in Refs.^42,59^. This aspect is worth future investigation, but it is not further considered here due to the reported time scale difference between the fast lipid diffusion (under $10\,\text{ms}$) and the slow protein rearrangements (from $100\,\mathrm {ms}$ to $10\,\mathrm s$^29,60^) as discussed in Refs.^40,43–45^. The pressure field, *p*(*r*, *z*), is represented in the main plot of Fig. 1c with *r* and *z* being the radial distance from the tubule axis and the axial distance from the polymer center, respectively. We build *p*(*r*, *z*) upon the assumption that GTP is abundant and readily hydrolyzed by a preassembled dynamin at a statistically steady rate. As anticipated, the power strokes build up tension along the chain length but keep it constant in the inner, statistically equilibrated turns. This provides the axial dependency of the pressure field shown in the lower red graph of Fig. 1c. On the other hand, the radial dependency of *p*(*r*, *z*) (upper blue graph of the same panel) is related to the internal tension of the chain via the inverse of the tubule radius and progressively fades to zero when the molecular structure of the helix reaches a maximum admitted curvature (around $3\,\mathrm {nm}$ in terms of tubule radius^14,33^, though the results are robust with respect to its exact value and to the shape of the decay function). Whenever tubule rupture is achieved, dynamin polymers disassemble^6^ and, accordingly, the pressure field vanishes. In this model, no depolymerization energy is deposited on the lipid membrane, in accordance with some recent evidence^18,32^. Such a diffuse model inherits the key geometrical aspects of the helix inasmuch as it depends on the polymerization length *H*, the pitch $h\approx 10\,\mathrm {nm}$^7,25,30,33^, the number of dimers per turn $N_d\sim \, 13$^14,30–33,41,61^, and the intensity of the averaged tangential traction exerted by the power units on each chain link, $F_{\tau }\approx \,2.5\,\mathrm {pN}$^24^ if not differently stated. These quantities appear in the pressure value $p(R_\text{in},0)=(N_d F_{\tau })/(h R_\text{in})$ attained at the center of the tubule when in its initial, undeformed configuration, $R_\text{in}$ being its radius, and are used in Fig. 1c to normalize the pressure as $p(r,z)=p^{\star }(r,z)\,\times \,p(R_\text{in},0)$.

In this work, we focus on systems characterized by open, axisymmetric portions of lipid tubules as they indeed represent the experimentally relevant scenario of long tubular bilayers pulled from a Giant Unilamellar Vesicle. In this setting, the vesicle, having a typical size well above the micrometer, acts as a lipid reservoir for the tubule. Moreover, the mechanical conditions imposed on the vesicles, for instance by micropipette aspiration, set the value of the surface tension $\gamma$ the tubule inherits from its boundaries. Following the ideas of the celebrated Canham^48^ and Helfrich^49^ model, the lipid bilayer (mesoscale) mechanics is mostly determined by the local curvatures of its midplane surface $\Gamma$, depicted in purple in the figures appearing hereinafter. An expression of the elastic Hamiltonian for symmetric, homogeneous composition bilayers, subjected to a constant surface tension $\gamma$, reads

1 $$\begin{aligned} \displaystyle \mathcal {H}_{CH}[\Gamma ] = \frac{k_b}{2} \int \limits _{\Gamma } \left( 2M \right) ^2 \, dS + k_{G} \int \limits _{\Gamma } G \, dS + \gamma \int \limits _{\Gamma } \, dS, \end{aligned}$$

where $k_b$ and $k_G$ are the bending rigidity and the Gaussian modulus, respectively, while *M* and *G* the mean and Gaussian curvatures of $\Gamma$. This picture is thought to hold as long as a scale separation persists between the curvature radii and the membrane thickness^62–64^, though there is evidence of its effectiveness in extreme-curvature tubules^63^. The second integral in Eq. (1) is characterized by an elusive behavior inherited from the Gauss–Bonnet theorem of differential geometry, relating the integral of the Gaussian curvature to the topology of the surface, and therefore accounts for a constant energy contribution as long as no topological rearrangement occurs. In this concern, lipid tubules preserve their topology throughout the whole constriction process, thus justifying the extensive use of these models in portraying the mechanical properties of their bilayer^17,18,20,22–26^. Nevertheless, the classical elasticity approach entails the impossibility to deal with topological transitions without recurring to surgical cut and paste operations on membrane patches^65^ and, consequently, cannot capture the energetics nor contemplate the Gaussian curvature-related forces involved in tubule fission. For these reasons, the recently proposed Ginzburg–Landau type free energy^50^, built on a diffuse interface description, is deployed to reproduce classical elasticity results in the first stages of constriction and account for Gaussian energy effects in a regularized way across neck cleavage. Such contributions are particularly relevant to the constitution of the energy barriers associated with topological rearrangements^50^ and also allow to envision possible mechanisms for their reduction, such as the catalytic effect of fusion peptides^66^. The adopted diffuse interface approach introduces a continuous phase field $\phi (\varvec{x})$, depicted by the contour plot on the lower half of Fig. 1b, which spans the entire three-dimensional domain $\Omega$ hosting the membrane, and distinguishes the internal and external environments, $\phi =1$ and $\phi =-1$, respectively. Thence, $\phi (\varvec{x})$ identifies the bilayer as the $\sim 5\,\mathrm {nm}$ diffuse region across the $\phi =0$ iso-surface, itself standing for $\Gamma$, and an effective, coarse-grained free energy is built thereupon.

In order to seize the effects of dynamin helices on these lipid tubules and portray the processes of constriction and fission, a forcing term, $I[\phi ]$, is suitably developed and introduced in the free energy of the system so as to properly spread the external pressure *p*(*r*, *z*) over the full width of the diffuse interface. The details of the mathematical model joining the contributions of the mean curvature (or bending) energy, $F_b[\phi ]$^46,47,67,68^, the Gaussian energy, $F_G[\phi ]$^50^, the surface tension energy, $F_{\gamma }[\phi ]$, and the interaction term are covered in Methods section.

A cylindrical lipid membrane of radius $R_\text{in}$ is straightforwardly found to minimize the elastic Hamiltonian in Eq. (1) when $R_\text{in}=\sqrt{k_b/(2 \gamma )}$. Though surface tension may sensibly vary depending on the specific experimental setting or physiological condition, it usually ranges between $10^{-6}$ and $5\times 10^{-4}\, \text{Nm}^{-1}$^18^. For the following results, if not differently stated, we adopt a typical value $\gamma =1.5\times 10^{-4}\,\text{Nm}^{-1}$. Upon choosing a reasonable set of physical parameters for the bilayer, consisting in $k_b=20\,k_BT$^69^, $k_G=-k_b$^70,71^, and membrane thickness $l_{me}=5\,\text{nm}$, we evolve the system from the undisturbed configuration of a microscopic-sized tubule ($1400\,\text{nm}$ long and with radius $R_\text{in}=16.6\,\text{nm}$) exploiting a maximum-dissipation-rate dynamics. Indeed, the coarse-grained free energy functional is minimized following a steepest gradient descent, also called Allen–Cahn dynamics (see Eq. (14)), which corresponds to an overdamped evolution with negligible inertial effects (the interested reader is encouraged to go through the details provided in “Methods” section). In order to set the actual time scale of the proposed dynamics, we perform a direct comparison with experiments on the kinetics of tubule deformation under external osmotic pressure^6^ (see “Methods” section as well as the Supplementary Information for additional details).

### The effect of polymerization length on the energetic requirements for fission

A hemifission intermediate stands along the fission pathway of lipid tubules and links the constriction phase to the severing completion, similarly to the hemifusion intermediate manifesting in the reversed, fusion process^18^. As experimentally determined^6,19,37^, the inner monolayer proceeds to fission before the outer one so that the impermeability of the membrane is preserved and the leak of enclosed substances prevented, thus momentarily realizing a single-layered, short, cylindrical micelle linking the now disjoined environments. The energy expenditure associated with this lipid reorganization was first estimated on a theoretical basis^17^, resulting in a spontaneous transition from the constricted configuration to the hemifission one upon reaching a critical membrane radius of $\sim 3\, \text{nm}$ at the site of tightest constriction, namely the membrane neck. An energy drop is thence estimated for completing fission, suggesting that the constriction alone could be sufficient for triggering the whole topological transition^17,18^. A minimal energy fission pathway has been numerically found with the aid of coarse-grained molecular dynamics approaches^45^, too, providing a non-negligible barrier of $\sim 30\, k_B T$ for rupturing the hemifission intermediate and completing fission. However, subsequent developments^40^ propose such a barrier not to be biologically relevant because of the specific simulation set-up, evidencing the persistence of challenges in the comprehension of such multiscale topological rearrangements. On the other hand, the copious experimental studies carried out over the last decade were able to delineate some recurring features of the fission process. Indeed, there is a common consensus on the existence of a critical neck radius, inasmuch as an unconstrained neck spontaneously proceeds towards fission whenever the inner luminal diameter or, equivalently, the neck radius approach the thickness of the lipid bilayer^36,41,72^.

In order to assess the proposed mesoscopic model and the role of the polymerization length *H* in determining severing effectiveness and efficiency, we perform a series of in silico experiments and compare some selected quantities with literature results. Hereinafter, we define the neck of the tubule as the site of maximum constriction. Furthermore, we define the critical state as the one with maximum elastic energy, $F_\text{e}=F_b+F_G+F_{\gamma }$, when it is followed by fission. Accordingly, there is no critical state when no fission occurs. It is worth specifying that multiple necks could form in general but, by definition, they all share the same minimum radius $R_n$.

**Figure 2:**
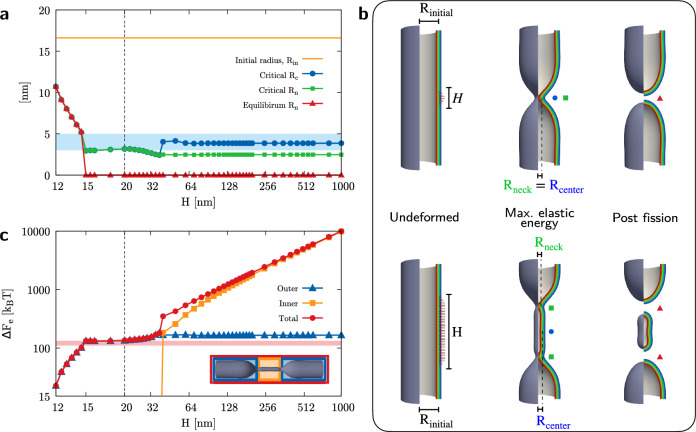
Effects of polymerization length on constriction and fission efficacy and efficiency. (**a**) Radius of maximum deformation sites, namely the neck radius $R_n$, at equilibrium (red line with triangles) and at maximum elastic energy (green line with squares), the latter preceding fission for $H\ge 15\, \text{nm}$, is depicted for increasing values of the coat height *H*. Critical radius at the coat center, $R_c$, is shown (blue line with circles), too. The initial radius $R_\text{in}=\sqrt{k_b/(2\gamma )}$ of the lipid tubule is displayed as a reference (orange line) and the range of theoretically and experimentally predicted critical neck and central radii is reported (light blue stripe)^17,36,41,72^. The value $H=20\,\text{nm}$ divides the abscissa in two different logarithmic scales so as to facilitate perception of all data. (**b**) Snapshots of tubule portions for different dynamin lengths ($H=20\,\mathrm {nm}$ above and $H=70\,\mathrm {nm}$ below) along the evolution. Specifically, from the first to the third columns, the system is depicted in its undeformed, critical, and severed states, respectively. Equilibrium neck radius (red triangle), critical neck radius (green square), and critical central radius (blue circle) are evidenced, too. (**c**) Maximum elastic energy content of the critical (when preceding fission) or equilibrium (when no fission occurs) configuration with respect to the initial, unperturbed one. The energy of the tubule (red circles) is shown for increasing dynamin heights together with the energy contributions from the central tubular region (orange square) and the outer flanks (blue triangle). This subdivision of the system is illustrated in the critical snapshot at the bottom of the graph (refer to the colored version of the article for better visibility). Experimentally estimated values for the minimal work expenditure of the optimal dynamin machinery^18^ is shown for reference (light red stripe). The value $H=20\,\mathrm {nm}$ divides the abscissa in two different logarithmic scales so as to facilitate perception of all data, whereas the ordinate follows a unique logarithmic scale.

Figure 2a shows the neck radii characterizing the equilibrium configurations attained for different values of *H* (red line with triangles). In particular, a null value here indicates that no neck exists at equilibrium, therefore determining the occurrence of a fission event. Along with that, the neck radii $R_n$ (green line with squares) and radii at the tubule center $R_c$ (blue line with circles) of the configurations with maximum elastic energy (critical ones, if preceding fission) are shown. Figure 2b depicts a collection of snapshots of tubule portions coated by a short, $20\,\mathrm {nm}$-long dynamin in the upper row and a longer, $70\,\mathrm {nm}$-long one in the lower row. The columns of this panel are organized so as to portray the evolution of the system from its undeformed state toward the severed one, passing through the critical configuration preceding fission. Equilibrium neck (red triangle), critical neck (green square), and critical central (blue circle) radii are evidenced. From the analysis in Fig. 2a, dynamin helices that barely complete a full turn around the tubule, i.e. with less than $\sim \, 18$ stalk dimers, do not complete fission but rather reach a maximum deformation that increases with the number of actively involved power stroke units. For longer coats, instead, fission is always observed and the critical radii that the tubules reach right before triggering the topological transition are found to be quite independent of the helix height, thus implying that an intrinsic critical length exists distinguishing whether the bilayer shall spontaneously proceed to fission or not. Notably, this same concept permeates the almost entirety of literature, where theoretical^17^ and experimental data^36,41,72^ identify the critical radius as $\sim 3\,\mathrm {nm}$ or comparable to the width of the bilayer. For the sake of comparison, this range is highlighted in Fig. 2a for a $5\,\mathrm {nm}$ thick membrane (light blue stripe). It is worth noticing that experimental estimates are often obtained through integral measurements, where the accessible observable is a sort of averaged radius of the putative cylindrical structure preceding fission, and, therefore, the slightly larger critical neck radius estimate ($\sim \,5\,\mathrm {nm}$) should be compared to the critical radius of the central region, $R_c$. As evident in Fig. 2a,b, indeed, long polymers manifest different behaviors between $R_n$ and $R_c$. This is related to the formation of a central, highly-constricted, tubular region enclosed by two distinct maximum deformation sites. In fact, the value assumed by $R_c$ for sufficiently long coats (large *H*) is consistent with the equilibrium radius of a tubular lipid membrane subjected to a tension modified by the dynamin pressure as $\bar{\gamma } = \gamma + N_d F_{\tau }/h$, as explained in Methods section, thus retrieving $R_c\approx \sqrt{k_b/(2 \bar{\gamma )}}=3.5\,\mathrm {nm}$.

The maximum elastic energy, $\Delta F_\text{e}$, is shown in Fig. 2c (red circles) versus the polymerization length *H*, taking as a reference the elastic energy of the unperturbed tubule. This maximum is achieved at equilibrium for coats shorter than $15\,\mathrm {nm}$, Fig. 2a, which do not reach fission. By increasing *H* and, therefore, the number of power stroke units, the elastic energy content increases. As for the radii, as soon as neck cleavage occurs, an elastic energy plateau ($\sim 130\,k_BT$) appears between $H=15\,\mathrm {nm}$ and $H=35\,\mathrm {nm}$, i.e. the amount of energy needed to sever the tubule is almost independent of *H*. This, together with the observation that particularly short polymers do not trigger fission, shows that an activation barrier ought to be overcome for it to occur and that, in the considered range of coat heights, fission proceeds in a similar fashion. Furthermore, this estimated activation energy ($\sim \,130\,k_BT$) is in astonishing agreement with the minimal effective amount of energy the GTP molecules must provide to the tubule for successful severing. As a matter of fact, literature data report the minimum number of GTPase cycles in the range between 15 and 18^18,36^ and an efficiency of $\sim \,37\%$ in effectively transferring to the membrane the $\sim 20\,k_BT$ of available energy per GTP, leading to the estimated $110\,k_BT$ to $130\,k_BT$, shown as a light red stripe in Fig. 2c. Noteworthy, these experimental estimates are performed on fairly short helices comprising about two rings since these structures are believed to represent the optimal machinery for tubule severing^19,33,40^ and, perhaps not by mere coincidence, are characteristic of clathrin-mediated endocytosis^60,73^. This, again, finds a correspondence in our numerical results (Fig. 2c) which indicate how chains with 1.5 to 3 loops (or $15\,\text{nm} \le H \le 30\,\text{nm}$) are more efficient than longer ones in achieving fission, in the sense that they are associated to a lower critical elastic energy and, therefore, a lower work expenditure. In this concern, long polymers are experimentally found to show a reduced efficiency or even impossibility^6^ to constrict and cleave tubule necks, unless GTP-enhanced disassembly takes place in advance, shortening the operating machinery^19^. Indeed, focusing on the long polymers branch of the plot in Fig. 2c, which means high values of *H*, the critical elastic energy increases linearly with *H* and soon reaches very high values. This regime is separated from the short helices plateau by a distinct step in $\Delta F_\text{e}$ taking place at $H\sim 35\,\mathrm {nm}$. In order to explain this evidence and bearing in mind the results in Fig. 2a,b, the critical elastic energy increment is split into two contributions: the energy of the tubule portions outside the neck (or necks), namely the outer flanks (blue triangles), and that of the constricted tubular (inner) region (orange squares) characterizing long helices. We find that the energy of the outer flanks retraces that of the whole tubule up until the end of the plateau, $H\sim \,35\,\mathrm {nm}$, and keeps constant therefrom. On the other hand, the creation of the inner tubular region is found responsible for the aforementioned step in the total elastic energy. When the length of the helix increases, so does, proportionally, the energy of the inner, highly constricted region.

### Determining fission site location

**Figure 3:**
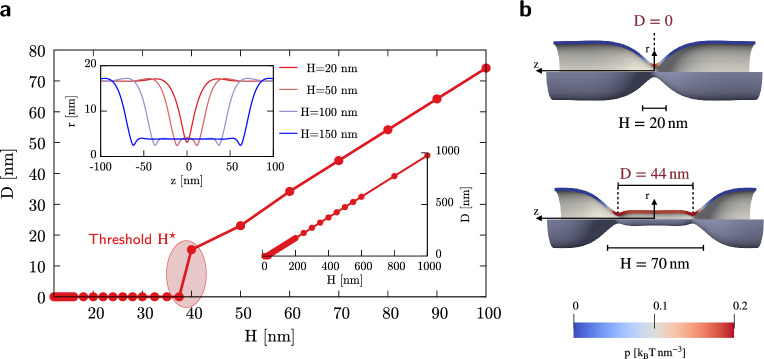
Fission site locations depend on polymerization length. (**a**) Distance *D* among critical necks for different polymerization lengths *H* of interest (main plot). The entire range of available results is shown in the inset on the right. *D* also depicts the distance between fission sites for those necks that are going to experience fission, i.e. for $H \ge 15\,\mathrm {nm}$. The inset on the left evidences the profiles characterizing the tubule deformation at the critical configuration for $H=20\,\mathrm {nm}$, $H=50\,\mathrm {nm}$, $H=100\,\mathrm {nm}$, and $H=150\,\mathrm {nm}$. The threshold polymerization length $H^{\star }$, identified at $\approx \, 40\,\mathrm {nm}$, differentiates the configurations leading to one or two fission sites. (**b**) Critical configurations for $H=20\,\mathrm {nm}$ and $H=70\,\mathrm {nm}$ evidencing the distance *D* between fission sites and the local pressure exerted by the GTP-activated dynamin action.

The analyses carried out so far show a different behavior between short, $H<40\,\mathrm {nm}$ (4 rungs), and long, $H>40\,\mathrm {nm}$, polymers, the most prominent difference being the formation of a single neck in the former case and two necks in the latter, see Fig. 3.

Two examples of critical configurations for short and long helices are shown in Fig. 3b, where the color contours depict the pressure exerted by the dynamin on the lipid bilayers. For long polymers, a constricted cylindrical structure separates the two necks and its axial extension increases linearly with *H*, as revealed by Fig. 3a. In fact, in this case, the necks form at a fixed distance from the coat edges.

Increasing *H* above a certain threshold value, $H^{\star }\approx 40\, \text{nm}$, the fission site shifts from the center of the dynamin coat to its periphery. These two behaviors were alternatively observed in experiments, see^36^ for fission at the center and^18,35^ for fission at the edges, and in molecular dynamics simulations^40,44^. In particular, Pannuzzo et al.^40^ discussed how this behavior is related to the polymer length. Although different explanations were proposed, ranging from the differential mechanical action of dynamin along its length^24^ to the greater stress due to the presence of an edge between coated and uncoated tubule regions^18^, our results endorse the idea that the location of the fission sites is determined by an intrinsic length related to the tubule elasticity. Specifically, the maximum constriction sites, namely the necks, anticipate the fission sites in the configuration where the elastic energy content is maximum (critical configuration), as also observed in experiments^32^.

**Figure 4:**
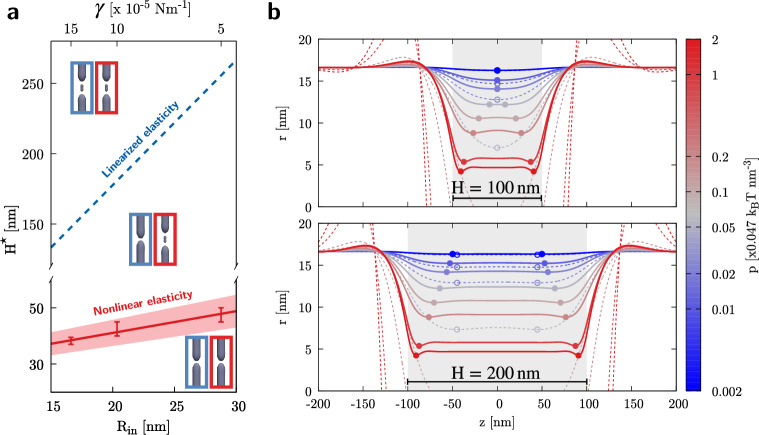
The role of nonlinear elasticity in fission pathway bifurcation. (**a**) Dependency of the threshold polymerization length ($H^{\star }$) on initial tubule radius (or membrane tension, with $k_b=20\,k_BT$). Linearized elasticity prediction, $H^{\star }_\mathrm{l.e.}=2\sqrt{2}\pi R_\text{in}$, is represented by the dashed blue line whereas nonlinear elasticity results are condensed in the solid red line, $H^{\star }=(25.6\pm 2.3)\,\text{nm} +(0.77\pm 0.11)R_\text{in}$, obtained by interpolation of the numerical results, red symbols (the shaded band represents the interpolation error while the vertical bars provide the confidence interval on the numerical data). Three zones are delineated in the phase diagram, where maximum constriction (linearized model, blue) and fission (nonlinear model, red) are predicted at the center or at the edge of the coat as summarized by the sketches. (**b**) Tubule deformation profiles ($R_\text{in}=16.6\,\mathrm {nm}$) under the effect of increasing pressure (see color bar): linear elasticity, dashed lines; complete Ginzburg–Landau elastic model, solid lines. Two different axial extensions (*H*) of the pressure distribution are reported: $H^\star<H=100\,\text{nm} < H^{\star }_\mathrm{l.e.}$, upper plot; $H^\star< H^{\star }_\mathrm{l.e.}<H=200\,\mathrm {nm}$, lower plot. Nonlinear elasticity anticipates the bifurcation of the fission pathway that starts occurring at smaller *H*. The circles highlight the maximum constriction sites (necks).

The fact that an intrinsic elastic length may explain the different fission scenarios is confirmed by the linearized Canham–Helfrich model, Eq. (19), which predicts an axial decay length, $L_\text{dec}$, proportional to the initial tubule radius, $L_\text{dec}=\sqrt{2} R_\text{in}$^74^. Actually, “Methods” section, the associated Green’s function is characterized by spatial oscillations exponentially decaying on the scale $L_\text{dec}$, see Fig. 6a below, which result into a single prominent dimple after convolution with the pressure of a short dynamin. To the contrary, a long coating leads to an almost complete cancellation of the oscillations except for the edges where two dimples survive, see Fig. 6b. In the linearized theory, the single dimple splits into two minima after the coating length exceeds the threshold $H^{\star }_\mathrm{l.e.}=2 \sqrt{2} \pi R_\text{in}$, see the blue dashed line in Fig. 4a.

Nevertheless, this prediction substantially differs from what we obtain from the present nonlinear simulations at critical conditions, whose data are shown in red in Fig. 4a with the bars providing the confidence interval. Following the motif of this work, we expect to find a direct relationship between the elastic parameters of the tubule and $H^{\star }$, as in the case of linearized elasticity. Indeed, varying $\gamma$ in a reasonable range^18^, we find that the nonlinear splitting height at critical conditions (threshold height $H^{\star }$) is determined by $R_\text{in}$ as shown by the red solid line in Fig. 4a. This delineates a phase diagram where fission of the tubule-dynamin assembly is expected to occur at the center or at the edge of the coat when below or above the threshold height $H^{\star }$, respectively. To further investigate the origin of the reduced threshold height for the nonlinear case, we compare the equilibrium deformation profiles, obtained through the minimization of the complete Ginzburg–Landau elastic free energy as well as through linearized elasticity, of tubules with $R_\text{in}=16.6\,\mathrm {nm}$ and subjected to radially-constant pressure fields with increasing intensities. The upper plot of Fig. 4b reports the case of $H=100\,\text{nm}$, expected to be below $H^{\star }_\mathrm{l.e.}$ but above the numerically found $H^{\star }$. The solutions taking into account nonlinear effects (solid lines) do superpose with linearized elasticity ones (dashed lines) for low intensities of the applied pressure (bluish colors), identifying a unique neck at the center of the coat (circles). However, they soon diverge when increasing the pressure intensity (reddish colors) and nonlinear elasticity results bifurcate into two distinct necks with increasing separation. Accordingly, when considering $H=200\, \text{nm}$, now above both $H^{\star }_\mathrm{l.e.}$ and $H^{\star }$, the structure of the equilibrium profiles is more consistent (lower plot of Fig. 4b) and the effect of nonlinear elasticity is limited to the containment of the deformation and the concurrent enlargement of the distance between necks.

### The time scale of fission

**Figure 5:**
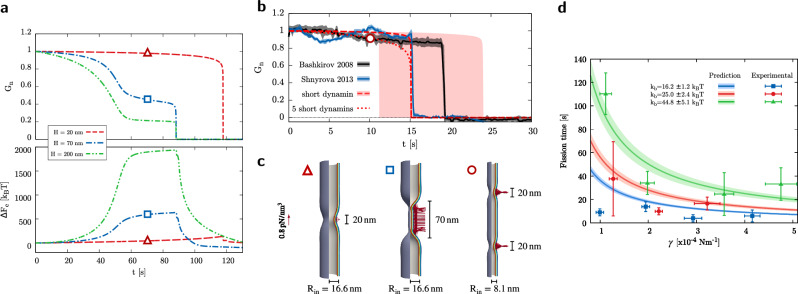
Time evolution of the system and fission time dependency on elasticity. (**a**) The Debye-corrected conductance of the internal lumen (*upper*) is measured during constriction and fission of $1.4\ \upmu m$-long tubules coated by differently polymerized dynamins ($H=20; 70; 200 \,\mathrm {nm}$, $F_{\tau }=2.5\,\mathrm {pN}$). Concurrently, the associated elastic energy change is shown (*lower*), evidencing the presence of an intrinsic energy barrier determining the closure of the lumen, i.e. null conductance. (**b**) Comparison of the time evolution of lumen conductance with experimentally available measurements^6,19^. The effect of a single short dynamin ($H=20\,\mathrm {nm}$) is depicted by the long dashed red line for $F_{\tau }=3.25\,\pm 0.75\,\mathrm {pN}$^24^, with the reddish area delineated by the uncertainty range of $F_{\tau }$. The effect of multiple (five) short dynamins, with $H=20\,\mathrm {nm}$, $F_{\tau }=3.25\,\mathrm {pN}$, and set $\sim 90\,\mathrm {nm}$ apart, is shown by the short dashed red line. The same elastic parameters characterize both the experiments and the numerical analysis, specifically $k_b=16\, k_B T$ and $\gamma =5 \times 10^{-4} \, \mathrm{Nm}^{-1}$, determining an initial tubule radius of $\sim 8\,\mathrm {nm}$. (**c**) Snapshots of the deformed tubules with arrows depicting the local dynamin force (per unit of volume) in the different conditions analyzed in panels a and b; scale arrow is $0.8\,\mathrm {pN}/\text{nm}^3$. From *left* to *right*, $16\,\mathrm {nm}$-radius tubule with a short dynamin, $16\,\mathrm {nm}$-radius tubule with a fairly long dynamin, and $8\,\mathrm {nm}$-radius tubule with five short dynamins, set $\sim 90\,\mathrm {nm}$ apart (only two shown here). Arrow scale, show on the left, is $0.8\,\mathrm {pN}\,\text{nm}^{-3}$. (**d**) Analytical prediction of the fission time, Eq. (2), compared with experimental results^18^ shown by the points with error bars. The solid, shaded lines represent the analytical predictions for the different values of $k_b$ together with their uncertainties, provided by Ref.^18^. Here, dynamin characteristics are $H=200\,\mathrm {nm}$ (experiments state $H>150\,\mathrm {nm}$) and $N_{d}\times F_{\tau } \approx 15\times 4\,\mathrm {pN}$. The actual time scale of the simulations is determined by a direct comparison with osmotic pressure induced constriction experiments^6^, as further discussed in “Methods” section and Supplementary Information.

To this point, we only focused on time-independent quantities like critical radii, activation energies, and fission site location. However, the time scale (and kinetics) of constriction and fission plays a fundamental role in the physiological function of dynamins. We therefore observe the time evolution of a $1.4\,\mathrm \mu m$-long tubule with initial radius $R_\text{in}\approx 16\,\text{nm}$ under the activity of differently polymerized dynamins. The chosen observables, shown in Fig. 5a, are the ratio of deformed-to-initial Debye-corrected lumenal conductance (normalized conductance $G_\text{n}$ shown in the upper plot, more details in the Supplementary Information) and the elastic energy change ($\Delta F_\text{e}$ in the lower plot). The occurrence of fission entails an abrupt decrease (to zero) of the lumen conductance, accompanied by a likewise steep decrease in the elastic energy. Prior to fission, along the constriction phase, the $G_\text{n}$ versus time curve remains substantially flat for short coats. On the contrary, it varies substantially when $H>H^{\star }$, i.e. where all dimers cooperate, as depicted by the blue and green dashed lines for $H=70$ and $200\,\mathrm {nm}$, respectively.

The normalized lumenal conductance is especially suitable for experimental comparison since it can be accurately measured along the constriction phase, at least for long tubules. Figure 5b shows such comparison for the case with $k_b=16\,k_BT$ and $\gamma =5\times 10^{-4}\,\text{Nm}^{-1}$, implying $R_\text{in}\approx 8\,\text{nm}$, using experimental results from the literature^6,19^, blue and black shaded lines. In these experiments, there is no direct control on the dynamin polymerization length, however, based on the present results (Fig. 5a and red long-dashed line—$H=20\,\text{nm}$, $F_{\tau }=3.25\,\text{ pN}$—in Fig. 5b), the modest reduction of the lumen conductance until severing takes place suggests the action of a short dynamin. Indeed, the authors themselves in Ref.^19^ suggest that dynamins might optimize their geometry for fission (attaining lower activation energies, as found in previous sections) by depolymerizing into separated collars made of few rungs and operating individually. This interpretation can be directly checked by comparing the conductance decrease induced by a connected, $70\,\mathrm {nm}$-long coat (dashed green line in Fig. 5a) with that of five $20\,\mathrm {nm}$-long collars, positioned $90\,\mathrm {nm}$ apart, see the short-dashed red line in Fig. 5b. Based on the data provided in Ref.^24^ we estimate the uncertainty in the value of $F_{\tau }$ as $F_{\tau }=3.25\pm 0.75\,\mathrm {pN}$, leading to the reddish band delimited by conductance profiles for $2.5\,\mathrm {pN}$ on the right and $4\,\mathrm {pN}$ on the left (single dynamin with $H=20\,\mathrm {nm}$). Panel c of Fig. 5 shows snapshots of the relevant region of the tubule along the constriction. The local forces exerted by dynamin on the lipid bilayer are shown as red arrows.

In another class of experiments^18^, the dynamins are significantly longer, $H>150\,\mathrm {nm}$. In this case, as shown in the upper inset of Fig. 3a (see, e.g., the blue line), the constricted region is basically cylindrical except for the two dimples at the edges. We have already discussed how the fission locations are determined by such dimples (necks). Nevertheless, a rough estimate of the constricted radii can be obtained by looking at the cylindrical portion. The advantage, here, is that the constriction dynamics of the cylindrical tubule can be analytically evaluated in exact form from the relaxation dynamics of Eq. (1), with the mobility estimated as discussed in the “Methods” section and Supplementary Information, forced by the pressure field exerted by the dynamin. As detailed in the Supplementary Information, we ultimately obtain an (approximate) analytical formula for the expected fission time, namely the time elapsed from the beginning of constriction to complete fission, which, for the case of $H>H^{\star }$, reads

2 $$\begin{aligned} t_\text{f}= \alpha (F_{\tau }) \frac{ R_\text{in}^2}{2 M_\text{sharp}} \left( \frac{\gamma +\bar{\gamma }}{{\bar{\gamma }}^2}\right) , \end{aligned}$$

where $\bar{\gamma }=\gamma +N_d F_{\tau }/h$, the system mobility is $M_\text{sharp}=3.57\,\mathrm{nm^4/(s}\ k_BT\mathrm{)}$, and $\alpha (F_{\tau })=1 +\text{exp}[(2.318\text{ pN}-F_{\tau })/0.805\text{ pN}]$ provides a small correction fitted on simulation data and vanishing for the upper range of $F_{\tau }=4\,\mathrm {pN}$. Equation (2) highlights the dependency of the fission time on elastic and mesoscopic parameters and its predictive capability is shown in Fig. 5d through a direct comparison with the already cited experimentally measured fission times for long dynamins ($H>150\,\mathrm {nm}$) in abundance of GTP^18^. These data are depicted by the symbols with errorbars for different combinations of $\gamma$ and $k_b$ while the theoretical predictions from Eq. (2) are shown as solid lines, with the shaded bands accounting for the experimental uncertainty on $k_b$. The dynamin geometry and traction in Eq. (2), which are not explicitly indicated in Ref.^18^, are chosen here such that $h=10\,\mathrm {nm}$ and $N_d\times F_{\tau }\approx 15 \times 4\,\mathrm {pN}$. Experimental fission times are reported to be independent of polymer length, at least in this scenario of $H>150\,\mathrm {nm}$, consistently with our present estimates.

It is worth mentioning that we also provide in the Supplementary Information a more general formula accounting for the fission times of both long and short dynamins.

## Discussion

Dynamin-driven fission of lipid tubules poses numerous challenges to both experimental and theoretical approaches inasmuch as it concurrently involves the molecular detail of the protein conformational changes and the full-scale elastic response of the lipid bilayer. In light of this, the mesoscopic model proposed in this work shall be understood as an attempt to effectively project the relevant physics of both these scales into a diffuse and continuous picture.

The results we have presented point to the central role played by the elastic interpretation of lipid membrane mechanics, which is found to emerge even at scales comparable to that of its thickness and whose nonlinear aspects have proven central for correctly predicting and explaining phenomena permeating the recent literature. In this concern, the diffuse representation of the interface and related (elastic) energy content was key to achieving a quantitative comprehension of the critical membrane geometry preceding fission. This was found to be in accordance with nourished experimental data^36,41,72^ and evidenced some recurring characteristics for a vast range of dynamin polymerization lengths (from tens to hundreds of nanometers). Beyond that, though the granular nature of the protein is lost along with the many molecular aspects, our results attest a surprising consistency of the adopted model in terms of mesoscale observables. In particular, this work hinges on a chain-inspired constriction mechanism activated by ratchet-like power strokes that take place between dynamin dimers^18,24,32,39^, eventually projected on the membrane as a diffuse inward-pointing pressure. Noticeably, this interpretation is compatible both with a free tilting of the PH domains, observed in experiments and crucial for fission^19,33,44^, and with an effort-free detachment between the polymer and the substrate, preventing the putative stabilization of super-constricted pre-fission states^4,33,40^. This is due to the absence of an explicit constraint between the dynamin and the lipid membrane, hence allowing them to adapt and cooperatively search for the optimal route to fission^19^.

Altogether, the proposed model is based on a reduced set of mesoscopic quantities that turned out to be sufficient for describing the relevant phenomenology. The polymerization length, for instance, distinguishes whether a dynamin is expected to successfully cleave a membrane neck and how efficiently this happens. In particular, we have delineated the existence of a minimal pathway to fission comprising a critical neck radius and geometry as well as a definite activation energy. This pathway is only accessible by dynamins with 1.5 to 3.5 rungs, suggesting this might be the reason why similar structures are believed to be optimal for fission^19^ and are found in in vivo processes, e.g. in clathrin-mediated endocytosis^60,73^. Longer coats, instead, trigger a bifurcation in the fission pathway coming along with an increased energy expenditure and a transition of the fission site from the center to the edge of the coat. Membrane elastic coefficients and the imposed surface tension, readily tunable by directly measuring the undeformed tubule radius, are found to control this bifurcation through nonlinear elasticity effects. Real-time conductance analyses^6,19^ are faithfully reproduced by the model, which also provides a fission time consistent with in vivo observations^60^. Moreover, the experimental dependence on the elastic parameters^18^ is reproduced and the optimality of short dynamin collars confirmed, eventually substantiating that the overall fission process can be described at the mesoscale level.

## Methods

### Ginzburg–Landau free energy functional for membrane elasticity

As discussed in the main text, the elasticity of the lipid membrane is characterized via an effective, coarse-grained, Ginzburg–Landau type of free energy. Based on a diffuse interface approach, we exploit this model for its ability to reproduce in a regularized way the effects of Gaussian curvature during topological transitions of the bilayer, otherwise inaccessible with classical, sharp elasticity descriptions like the Canham–Helfrich model^48,49^. The contributions of bending energy, $F_b[\phi ]$^46,47,67,68^, the Gaussian energy, $F_G[\phi ]$^50^, and the membrane tension energy, $F_{\gamma }[\phi ]$, were extensively discussed on both a physical and a mathematical basis in Bottacchiari et al.^50^, and alternative formulations for the bending and tension energies were proposed in literature^75,76^. For the reader convenience, we restate here their expressions appearing in the elastic free energy, $F_\text{e}[\phi ]$, as

3 $$\begin{aligned} \displaystyle F_\text{e}[\phi ] =&F_b[\phi ]+F_G[\phi ]+F_{\gamma }[\phi ], \end{aligned}$$

4 $$\begin{aligned} F_b[\phi ]=&\frac{k_b}{2} \frac{3}{2\sqrt{2}\epsilon ^3} \int \limits _{\Omega } \psi _{b}^2 \, dV, \end{aligned}$$

5 $$\begin{aligned} F_G[\phi ]=&k_{G} \frac{35}{16\sqrt{2}}\epsilon ^3 \int \limits _{\Omega } \psi _{G} \, dV \,, \end{aligned}$$

6 $$\begin{aligned} F_{\gamma }[\phi ]=&\gamma \frac{3}{2\sqrt{2}\epsilon } \int \limits _{\Omega } \left[ \frac{1}{4} (\phi ^2-1)^2 + \frac{\epsilon ^2}{2}(\varvec{ \nabla } \phi )^2 \right] \, dV, \end{aligned}$$

with $\psi _b$ and $\psi _G$ two functions of $\phi$ expressed as

7 $$\begin{aligned} \psi _b(\phi )=\phi (\phi ^2-1)-\epsilon ^2\nabla ^2\phi , \end{aligned}$$

8 $$\begin{aligned} \begin{aligned} \psi _G(\phi )&=\dfrac{ \varvec{\nabla } |\varvec{\nabla } \phi |^2 \cdot \varvec{\nabla } |\varvec{\nabla } \phi |^2}{2} - (\varvec{\nabla } |\varvec{\nabla } \phi |^2 \cdot \varvec{\nabla } \phi ) \nabla ^2 \phi \; +\\&\quad + \; |\varvec{\nabla } \phi |^2 \bigg [(\nabla ^2 \phi )^2 +\varvec{\nabla } \phi \cdot \varvec{\nabla } \nabla ^2 \phi - \dfrac{\nabla ^2 |\varvec{\nabla } \phi |^2}{2}\bigg ]. \end{aligned} \end{aligned}$$

In this model, $\epsilon$ is a parameter related to the width of the diffuse interface separating the $\phi =-1$ and $\phi =1$ regions of the space $\Omega$. In the limit of vanishing $\epsilon$, the diffuse free energy in Eq. (12) converges to the sharp Canham-Helfrich functional appearing in Eq. (1). The Canham–Helfrich model uniquely depends on a macroscopic length scale, such as perhaps the typical lateral extension of the membrane or curvature radius. However, the occurrence of a fission event—or, in general, the proximity to a topological change—brings into play an additional physical scale that pertains to the width of the bilayer, $l_{me}\approx 5\,\mathrm {nm}$. Therefore, as detailed in Ref.^50^, we introduce this quantity in the phase field energy by requiring a matching between the diffuse interface thickness, identified by $l_{pf}\approx 6\epsilon$, and that of the actual bilayer. As a result, we set $6\epsilon =5\,\mathrm {nm}$.

### Interaction energy and dissipative dynamics

The effect of dynamin pressure on the lipid tubule is modeled via a suitable interaction term, $I[\phi ]$, appearing in the free energy of the system. In order to properly spread the external pressure over the full width of the diffuse interface, we define a mediating term, $h(\phi )$, as

9 $$\begin{aligned} h(\phi )=\frac{3}{4} \left( \phi - \frac{1}{3} \phi ^3 \right) , \end{aligned}$$

therefore obtaining

10 $$\begin{aligned} I[\phi (\varvec{x})]=\int \limits _{\Omega } h(\phi (\varvec{x}))\, p({\varvec{x}})\, dV. \end{aligned}$$

The nature of this forcing term might be explained via a comparison with classical, sharp elasticity theory. The elastic reaction force (per unit of surface) far from topology changes is expressed as^77^

11 $$\begin{aligned} \varvec{f}_\text{e} = -[2\gamma M - 4 k_b M(M^2-G)-2k_b \Delta _{\pi }M]\varvec{n}_{\Gamma }, \end{aligned}$$

with $\varvec{n}_{\Gamma }$ the surface normal to $\Gamma$ and $\Delta _\pi$ its associated Laplace-Beltrami operator. Notably, the differential terms within the square brackets in the right-hand side of Eq. (11) correspond to the variational derivative of Eq. (1), required to vanish at equilibrium in the so-called shape equation. When subjected to external pressure distributions, $p_\text{ext}$, classical elasticity identifies the new equilibrium as the configuration where $\varvec{f}_\text{e}+p_\text{ext}\varvec{n}_{\Gamma }=0$. Analogously, it is possible to show that the elastic reaction forces (per unit of volume) arising from the diffuse interface model are expressed as^50^

12 $$\begin{aligned} \varvec{f}_\text{e}(\phi )=\frac{\delta F_\text{e}[\phi ]}{\delta \phi }\varvec{\nabla }\phi , \end{aligned}$$

with $\delta F_\text{e}[\phi ]/\delta \phi$ the functional derivative of the elastic energy in Eq. (3). The gradient of the phase field defines the unit normal to its iso-surfaces as $\varvec{\nabla }\phi /|\varvec{\nabla }\phi |=\varvec{n}$, hence $\left. \varvec{n}\right| _{\phi =0}=\varvec{n}_{\Gamma }$ is the unit normal to membrane midplane. Now, evaluating

13 $$\begin{aligned} \varvec{f}_\text{ext}(\phi )=\frac{\delta I[\phi ]}{\delta \phi }\varvec{\nabla }\phi =p\,\frac{3}{4}(1-\phi ^2)\varvec{\nabla }\phi , \end{aligned}$$

minimization of the complete free energy functional, $F[\phi ]=F_\text{e}[\phi ]+I[\phi ]$, leads to $\varvec{f}_\text{e}(\phi )+\varvec{f}_\text{ext}(\phi )=0$. In other words, $\varvec{f}_\text{ext}(\phi )$ is the external force field spread over the diffuse interface width, which is the equivalent in the diffuse interface context of the pressure applied to the sharp model. Indeed, as $\epsilon /R_\text{in} \rightarrow 0$, we obtain in the weak limit the distribution $3/4\,(1-\phi ^2)|\varvec{\nabla } \phi | {\mathop {\longrightarrow }\limits ^{\mathcal {W}}} \delta (d(\varvec{x}))$, where $\delta (d)$ is the Dirac delta function of the signed distance from $\Gamma$.

These results show that the equilibrium configurations of a perturbed bilayer found by classical sharp models or by the proposed diffuse interface approach are equivalent in the small thickness limit. This correspondence can be extended to the whole dynamics since, under the same assumptions, the Allen–Cahn dynamics of the phase field, see Eq. (14) below, can be shown to correspond to the sharp interface evolution of the membrane^78^. The Allen–Cahn dynamics, also known as gradient descent or maximum dissipation rate dynamics, reads

14 $$\begin{aligned} \frac{\partial \phi }{\partial t}= -M_\text{pf} \frac{\delta F[\phi ]}{\delta \phi } =-M_\text{pf} \left( \frac{\delta F_\text{e}[\phi ]}{\delta \phi }+\frac{3}{4}(1-\phi ^2)\,p \right) , \end{aligned}$$

where $M_\text{pf}$ is the mobility of the system. As shown in the “Results” section and Supplementary Information, the Allen–Cahn dynamics provides physically relevant results also for finite membrane thickness, where it allows to describe topological rearrangements.

Equation (14) can be given a straightforward physical interpretation. After multiplying by $\varvec{\nabla }\phi$ and using Eqs. (12) and (13), the equation is rearranged as $\varvec{f}_\text{e}+\varvec{f}_\text{ext}=-1/M_\text{pf} \,\partial \phi / \partial t \varvec{\nabla }\phi$, which, on account of the definition of displacement velocity $\varvec{u}=-\partial \phi /\partial t\,\varvec{\nabla }\phi |\varvec{\nabla }\phi |^{-2}$, is rewritten as

15 $$\begin{aligned} \varvec{f}_\text{e}+\varvec{f}_\text{ext}+\varvec{f}_\text{visc}=0, \end{aligned}$$

where $\varvec{f}_\text{visc}=-\nu \varvec{u}|\varvec{\nabla }\phi |^2$ is a friction force linear in the velocity and localized by $|\varvec{\nabla }\phi |^2$ on the surface $\Gamma$, with $\nu = 1/M_\text{pf}$ the friction coefficient.

A direct comparison with experimental data concerning real-time constriction of a lipid tubule under the influence of osmotic pressure^6^ allows us to estimate $M_\text{pf}=4.04 \,\mathrm{nm^3/(s\ }k_BT\mathrm{)}$ which yields $M_\text{sharp}=3\epsilon M_\text{pf}/\sqrt{8} =3.57\,\mathrm{nm^4/(s\ }k_BT\mathrm{)}$ for the sharp interface counterpart (see Supplementary Information for the complete discussion).

### Constriction of cylindrical structures as a modified membrane tension

In the long coats branch of Fig. 2a, the central region of the constricted tubule takes the shape of a cylinder with axial extension *L* and a radius $R=R_c$, substantially independent of *H*. Neglecting edge effects ($H/L_\text{dec}\gg 1$), this cylindrical, highly constricted region is the result of an axially-uniform pressure $p(r)=(N_d F_{\tau })/(h\,r)$. Thereby, the Hamiltonian of the cylindrical patch reads

16 $$\begin{aligned} \mathcal {H}_\text{sharp} = k_b \frac{\pi L}{R}+\gamma 2 \pi R L+ \frac{N_d F_{\tau }}{h} 2\pi R L, \end{aligned}$$

revealing that dynamin constriction effectively results in a modification of the surface tension as $\bar{\gamma }=\gamma +N_d F_{\tau }/h\,$. By minimizing Eq. (16) with respect to *R*, we find the constricted equilibrium radius

17 $$\begin{aligned} R_c=\sqrt{\frac{k_b}{2\left( \gamma +\frac{N_d F_{\tau }}{h}\right) }}=\sqrt{\frac{k_b}{2\bar{\gamma }}}. \end{aligned}$$

Noticeably, since $N_d F_{\tau }/h\gg \gamma$ and since the estimated rupture tension of lipid bilayers is well below $\bar{\gamma }$^6^, directly applying a membrane tension would not allow to achieve such intense constrictions.

### Linearized elasticity predictions

**Figure 6:**
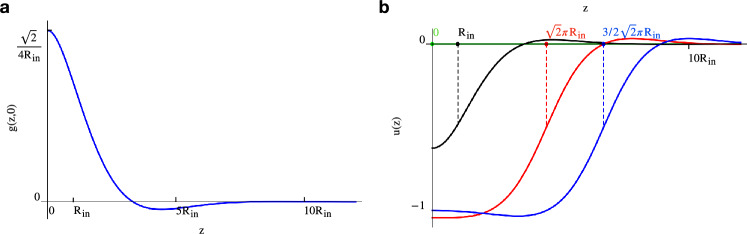
Linearized elasticity analyses. (**a**) Graph of the Green function, expressed in Eq. (20), for the linearized elastic problem in Eq. (19). (**b**) Deformations obtained under a squared perturbation of unitary intensity and different widths, see Eq. (21). The green, black, red, and blue curves correspond to perturbation of widths 0, $2R_\text{in}$, $2\sqrt{2}\pi R_\text{in}$, and $3\sqrt{2}\pi R_\text{in}$, respectively. The red curve marks the separation between the deformations with a unique maximum constriction site, or neck, at $z=0$ (like the black curve) and those with two distinct necks at $z\ne 0$ (like the blue curve). Only the positive semi-axis $z>0$ is shown in the two panels since the problem is symmetric with respect to $z=0$.

Linearized elasticity is crucial for determining the origin of fission pathway bifurcation in long coats, i.e. the transition from a single neck to multiple ones, coming along with the formation of an enclosed, constricted, and cylindrical structure. First, an axisymmetric expression for the perturbed shape equation of the elastic Canham–Helfrich functional in Eq. (1) is retrieved^79^ and suitably parametrized in terms of the distance *r*(*z*) from the symmetry axis and the angle $\psi (z)$ between the *r*-axis and the tangent to the midplane profile. This reads

18 $$\begin{aligned} 2r^2 s^3 \psi '''+8r^2s^2c\,\psi '' \psi ' +r\,s(2c^2-s^2)\psi '^3 +4r\,c\,s^2\psi '' +r\,s(4-7s^2)\psi '^2 -(3c^2-1)s\,\psi '-2r^2s\,\psi ' \frac{\gamma }{k_b}-2r\,s\,\frac{\gamma }{k_b} +\frac{1+c^2}{r}s =-2r^2\frac{p}{k_b}, \end{aligned}$$

with $s=\sin {\psi }$, $c=\cos {\psi }$, and *p* the imposed pressure distribution, itself function of *r* and $\psi$. This differential equation is then expanded in a weakly perturbed, nearly cylindrical approximation, where $p=\alpha \, \bar{p}$, $r(z)=R+\alpha u(z)$, $\psi \sim \pi /2$, $\bar{p}$ is *O*(1), and $\alpha$ is small. In the limit for $\alpha \rightarrow 0$, Eq. (18) provides $R=R_\text{in}=\sqrt{k_b/(2\gamma )}$. Then, retaining only the linear terms in $\alpha$, the above equation greatly simplifies to

19 $$\begin{aligned} R_\text{in}^4\,u^{(4)} \,+\,u = \bar{p}\, \frac{R_\text{in}^4}{k_b}, \end{aligned}$$

where the superscript in $u^{(4)}$ refers to the differentiation order with respect to *z*. The Green’s function of the unforced equation, $\bar{p}=0$, under the boundary conditions of finite and flat perturbation far from the center of the tubule, $z\rightarrow \pm \infty$, is thence

20 $$\begin{aligned} g(z-y)=\frac{\sqrt{2}}{4R_\text{in}}e^{-\frac{|z-y|}{\sqrt{2}R_\text{in}}}\left( \cos {\frac{|z-y|}{\sqrt{2}R_\text{in}}} + \sin {\frac{|z-y|}{\sqrt{2}R_\text{in}}} \right) , \end{aligned}$$

with *y* the position of the disturbance. This equation reveals that $g(z-y)$ is an even function and that the lipid tubule response to a weak perturbation decays exponentially with an elastic relaxation length $\sqrt{2}R_\text{in}$, as shown in Fig. 6a. Moreover, the trigonometric functions in Eq. (20) provide the oscillations in the membrane deformation which are are now shown to be at the origin of the fission site splitting.

Indeed, the deformation resulting from a constant, rectangular-windowed pressure, $\bar{p}(z)=-k_b/R_\text{in}^4 \,\Pi (z/H)$ with $\Pi (z/H)$ the rectangular function of width *H*, may be computed as the convolution

21 $$\begin{aligned} u(z)=\int \limits _{L}g(z-y) \bar{p}(y)\,dy. \end{aligned}$$

The resulting deformation has infinite stationary points. Analytically finding the maximum constriction sites, namely the necks, is not an easy task, since one should start from solving

22 $$\begin{aligned} u'(z)=-\frac{k_b}{R_\text{in}^4}\int \limits _{-H/2}^{H/2}g'(z-y)\,dz= \int \limits _{-H/2-z}^{H/2-z}g'(x)\,dx=0 \quad \Rightarrow g(H/2-z_c)=g(H/2+z_c), \end{aligned}$$

for $z_c$ the stationary points. Nonetheless, we are only interested in predicting the value $H^{\star }_\mathrm{l.e.}$ above which the maximum constriction sites are more than one and located at $z\ne 0$. Figure 6b provides some examples of deformed profiles for different perturbation widths *H*. As evident from the sequence of green-black-red-blue curves, while increasing *H* the minimum of the plot shifts from $z=0$ to $z>0$. This suggests that there is a smooth transition condition where the minima are very close to $z=0$. Under this hypothesis, we expand the above equation for $z_c$ about $z=0$ and obtain, in the limit $z_c \rightarrow 0$,

23 $$\begin{aligned} 0+z_c\left[ -2 \cos \left( \frac{H}{2\sqrt{2}R_\text{in}} + \frac{\pi }{4} \right) +2\sin \left( \frac{H}{2\sqrt{2}R_\text{in}} + \frac{\pi }{4} \right) \right] + O(z_c^2) = 0. \end{aligned}$$

Equation (23) is solved at the zeroth order in $z_c \rightarrow 0$ by all values of *H*. This is the trivial solution since the deformation is always an even function and admits a null derivative in $z=0$ thereof. When looking for stationary points close but not equal to zero, we need to solve for the first order in $z_c\rightarrow 0$. This requires $H=2\sqrt{2}\pi R_\text{in} k$, with *k* an integer number. The smallest positive value of this set is what we called threshold height for the linearized elasticity problem, therefore

24 $$\begin{aligned} H^{\star }_\mathrm{l.e.}=2\sqrt{2}\pi R_\text{in}. \end{aligned}$$

### Numerical implementation

Equation (14) is numerically implemented with a FFT-based spectral differentiation on cell-centered grids and evolved through a semi-implicit Euler single-step scheme. This provides sufficient accuracy especially when evaluating the high-order derivatives arising from Eq. (8), as discussed in Ref.^50^. All axisymmetric simulations (apart from the one with $R_\text{in}=29\,\mathrm {nm}$ in Fig. 4a) where performed on a $30\,\text{nm}\times 1400\,\text{nm}$ computational domain with a $108\times 5040$ homogeneous grid. The initial configuration represents the undeformed cylindrical tubule as $\phi (r,z)=\tanh \left( (R_\text{in}-r)/(\sqrt{2} \epsilon ) \right)$.

### Supplementary Information

Supplementary Information.

## Data Availability

The main data are provided within the manuscript. The raw data from which figures have been generated is available from the corresponding author on reasonable request.
