# Supplementary material for: Mesoscopic elasticity controls dynamin-driven fission of lipid tubules
Source: Sci Rep. 2024 Jun 18;14:14003. doi: 10.1038/s41598-024-64685-2 (PMC11189461; doi:10.1038/s41598-024-64685-2)
Supplement: Supplementary file 1 — Supplementary Information. [file 41598_2024_64685_MOESM1_ESM.pdf]

Mesoscopic elasticity controls dynamin-driven fission of lipid tubules
Supplementary Information

Marco Bussoletti, Mirko Gallo, Matteo Bottacchiari, Dario Abbondanza, and Carlo Massimo Casciola*
Department of Mechanical and Aerospace Engineering, Sapienza University, Rome, Italy
(Dated: May 18, 2024)

Construction of the mesoscopic pressure field for dynamin

In order to construct a mesoscale interpretation of the dynamin action on axisymmetric lipid tubules, eventually defining $p(r, z)$ of the main text, we ought to start from the structure of the polymer and the molecular level activities characterizing the GTP-consuming conformational changes. The prototypical dynamin monomer comprises five domains [1–4] organized as follows. A rigid stalk connects to the membrane-binding pleckstrin homology domain (PH) via flexible linkers and to the outwards-facing GTPase domain (G) through a bundle signaling element (BSE). The latter links to the stalk and G domains via the flexible joints hinge 1 and hinge 2, respectively, while an unstructured C-terminal, the proline-rich domain (PRD), interacts with endocytic recruiting proteins. Stalks are found to crisscross associate via interfaces 2 in an anti-parallel dimer, itself constituting the building block of the helix, and consequently tetramerize via interfaces 1 and 3 [3, 5], these being associated to relatively soft bending modes [1, 6]. The geometry and associated mechanics of the resultant helix are therefore defined by these interfaces and oligomerization may only take place on compliant, high-curvature, and quasi-cylindrical lipid substrates.

Supposing the helix to be initially preassembled on the cylindrical substrate in the absence of GTP, we may model the dynamin polymer as a chain, where each link stands for one anti-parallel stalk dimer. The PH domains of each link are thence anchored to the underlying bilayer and are responsible for the force transmission onto the tubule [7]. Concurrently, G domains of adjacent rungs face each other on the outer layer of this chain-like structure while bundle signaling elements (BSEs) flexibly connect them to the stalk links. Overall, these domains are coherently distributed in concentric cylinders [8], where the lipid-protein interfaces occupy the innermost layer, oppositely to G domains. Upon GTP addition, dimerization of apposed G domains is favored and, consequently, GTP hydrolysis triggers the conformational change consisting in an angular contraction between the BSE and G domains about the flexible hinge 2. Eventually, the hydrolyzed nucleotide is released, thus completing the cycle that determines the GTP-consuming, ratchet-like power stroke [6, 9–11]. The latter mechanism results in an attraction force between the interested links of the adjacent rungs and, given the specific geometry of G domains cross-dimerization, translates into a force component parallel to the tubule axis, which is readily discharged on the tightly packed helix, and, more interestingly, a force component tangential to the cylindrical structure and normal to the tubule's axis. A joint effort of molecular dynamics, structure-based simulations [12] and single-molecule FRET observations provided a measure for this latter component, resulting in a tangential force $F_\tau \approx 2.5$ to $4\,pN$ acting so as to make the dimer slide in a helix-constriction sense [6]. Noteworthy, the elastic response of the helix to changes in curvature and torsion throughout tubule constriction is found to be subdominant with respect to the energy cost for deforming the membrane [6]. Altogether, these features substantiate the mesoscale interpretation of the dynamin polymer as a tightly packed helical chain coating the lipid tubule on a H-wide region, qualitatively depicted in Figure 1a of the main text and Figure S.1a here. Defining z the axial coordinate of the tubule with zero reference at the middle of the coat and r the radial distance from the latter axis, each element of the chain occupies a width comparable to the helical pitch $h \approx 10\,nm$ in the z-direction, rationalized by the strict packing visible in Cryo-EM experiments combined with 3D map reconstructions [2, 7, 13, 14], whilst having a $\sim 15\,nm$ thickness on the orthogonal plane spanned by r. Due to the relatively large thickness of the dynamin coat, the external radii and pitch of the helix as well as the overall height H of the coat experience negligible deformations for our purposes [15, 16]. Furthermore, the discussed conformational changes come along with a tilting of the stalk [11], thus reducing the distance between consecutive motors and keeping unaltered the external geometry during constriction. As a matter of fact, the number of power stroke units per rung does not change sensibly during the whole constriction process and assesses on a value of $N_d \approx 13$ [2, 3, 5, 10, 13, 15, 16].

We introduce a helical curve following the ideal locations of the stalk dimers (black curve in Figure S.1a and b). This is defined by a $h \approx 10\,nm$ pitch and has a $R \approx (r_\mathrm{m} + 8\,nm)$ radial distance from the z-axis of the tubule, whose midsurface radius is r_m. This curve is parametrized by its arc length $s \in [0, L]$ and defines a Frenet-Serret orthonormal

* carlomassimo.casciola@uniroma1.it

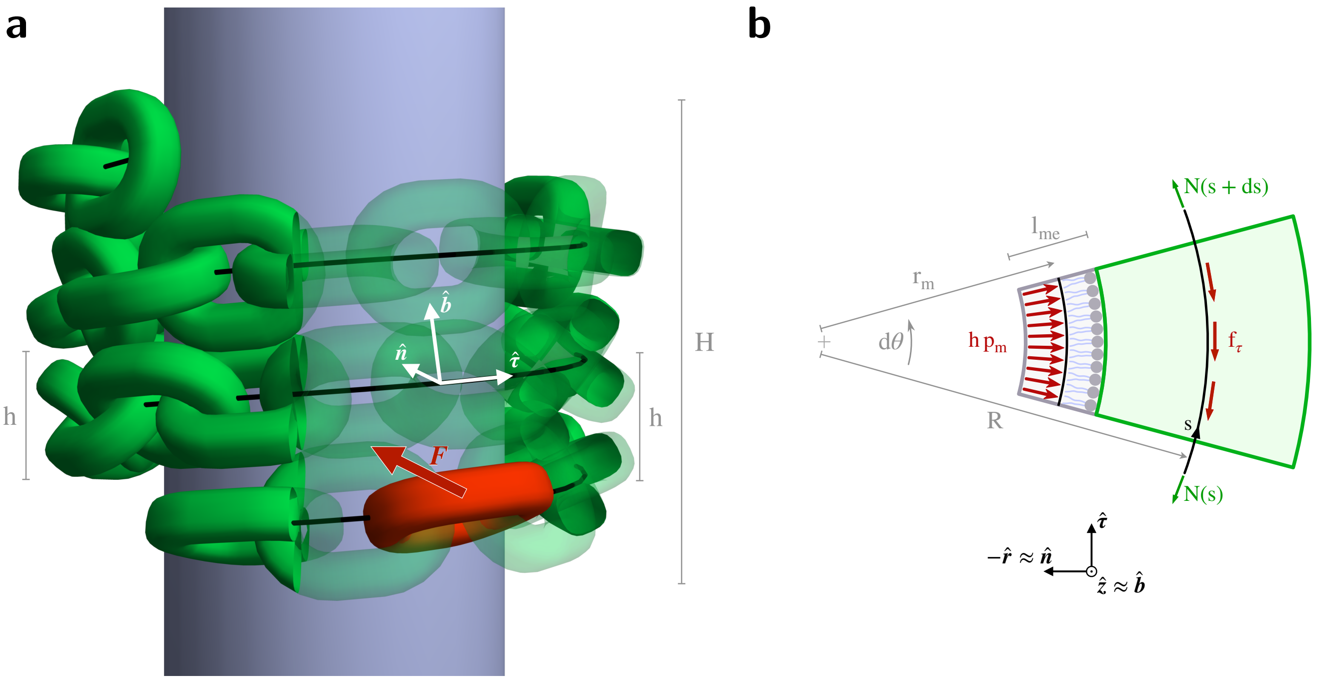

FIG. S.1. The mesoscopic effect of dynamin on the lipid tubule. (a) The dynamin polymer has a helical structure and is here represented with a (green) chain coiled around the (purple) lipid tubule in the initial, undeformed configuration. The black helical curve, parametrized by its arc length s, passes through the hypothetical locations of the stalk domains and defines the Frenet-Serret orthonormal basis shown in white. Each chain link stands for a dynamin dimer and, therefore, two oppositely oriented G-domains stick out from each. The G-domains of adjacent rungs interact in presence of GTP and produce an attractive force (the red arrow $\boldsymbol{F}$) between the interested links (one of which is shown in red). This force is statistically present in every pair of oppositely facing chain elements of adjacent rungs with the appropriate inclination, see [6] for a detailed explanation. (b) In order to model the pressure experienced by the lipid bilayer during dynamin constriction, we sketch the structure of a short (angular span is $d\theta$ and radius R) helix branch where the dynamin chain and the interested membrane region do balance. Under the hypotheses detailed in the text, each piece of the helical curve (black curve) is subjected to a tangential tension $N(s)$ and a distributed tangential load f_τ representing the diffuse action of the power strokes. Finally, the diffuse chain (shown in green) is balanced via a radial force per unit length exerted by the membrane reaction $h\,p_{\mathrm{m}}$ and thought of as acting on its r_{m}-radius midsurface. The assumption of quasi-cylindrical structure leads to $\hat{\boldsymbol{n}} \approx -\hat{\boldsymbol{r}}$ and $\hat{\boldsymbol{b}} \approx \hat{\boldsymbol{z}}$, with z and r the axial and radial coordinates of the axisymmetric system.

basis composed of the tangential $\hat{\boldsymbol{\tau}}$, normal $\hat{\boldsymbol{n}}$, and binormal $\hat{\boldsymbol{b}}$ unit vectors. The chain is able to bear axial tension ($N(s)\hat{\boldsymbol{\tau}}$), but no relevant bending response is expected in the range of deformations it experiences [1, 6]. Moreover, a mechanical balance of forces may be assumed since inertial and damping effects are negligible for the chain. Based on this, we can evaluate the axial load the chain experiences during constriction and to which the enclosed bilayer shall react through a radial pressure p_{m}. Noticeably, only radial forces are expected from the bilayer since shear stresses due to viscous effects on the bilayer surface are not included in this analysis, following the remarked [17, 18] time-scale separation between the fast lateral reorganization of lipids ($\sim 10\,ms$) and the slow dynamin kinetics (~ 0.1 to $1\,s$ [1]). Under the hypotheses that the tubule is axisymmetric and quasi-cylindrical (*i.e.* r_{m} varies slowly along z) and recognizing the modest inclination of the helix ($h/(2\pi R) \ll 1$), we approximate each rung as horizontal and enforce $\hat{\boldsymbol{n}} \approx -\hat{\boldsymbol{r}}$ and $\hat{\boldsymbol{b}} \approx \hat{\boldsymbol{z}}$. All these considerations imply that R only slightly changes along the helix length. The force $\boldsymbol{F}$ exerted by the power stroke on a single link is depicted by the red arrow in Figure S.1a. The first homogenization step consists of distributing the tangential components F_τ of these forces as a force per unit length along s, namely $f_\tau = F_\tau N_d/(2\pi R)$ in Figure S.1b, with N_d the number of dimers per rung. The sketch in Figure S.1b assists in the expression of the mechanical balance for a short branch of the helix (or chain), where r_{m} and R are essentially constant. Indicating with $d\theta$ the infinitesimal angular span of this branch and with ds its extension on the arc length, the balance reads

$$\hat{\boldsymbol{r}}: \qquad -N(s)\sin\frac{d\theta}{2} - N(s+ds)\sin\frac{d\theta}{2} + \int_{-d\theta/2}^{d\theta/2} h\,p_{\mathrm{m}}(s)\,r_{\mathrm{m}}\cos\alpha\,d\alpha = 0\,, \tag{S.1}$$

$$\hat{\boldsymbol{\tau}}: \qquad N(s+ds)\cos\frac{d\theta}{2} - N(s)\cos\frac{d\theta}{2} - \int_{-d\theta/2}^{d\theta/2} f_\tau(s) R\cos\alpha\, d\alpha \, = 0\,. \tag{S.2}$$

We recall that the force component along $\hat{\boldsymbol{z}}$ is straightforwardly balanced by the strict packing of the chain. Since $d\theta$ is infinitesimal, $p(s)$ and $f_\tau(s)$ vary slowly along s, and $ds = R\,d\theta$, at the first order of approximation we get

$$-N(s) + r_\mathrm{m}\, h\, p_\mathrm{m}(s) \, = 0 \qquad \text{and} \qquad \frac{dN(s)}{ds} - f_\tau(s) \, = 0\,. \tag{S.3}$$

Noticeably, in Equation S.1 the radial force stemming from the axial tension $N(s)$ is balanced by the force per unit length sustained by the lipid bilayer (here thought of as exerted by the membrane midsurface). This latter consists of the pressure p_m times the height of the contact region of each dimer, approximated as h. Since each chain link presents two oppositely oriented G domains, the inner turns of long polymers are statistically in balance. As a result, $f_\tau(s)$ is different from zero only in the rungs at the edge of the helix and these effectively build up the tension that is thereof maintained constant in the inner branches of the chain. Altogether, we solve for the axial tension and membrane pressure as

$$\begin{cases} N(s) = \dfrac{N_d F_\tau}{2\pi R_\mathrm{ext}} s\,; & p_\mathrm{m}(s) = \dfrac{N_d F_\tau}{2\pi R_\mathrm{ext}} \dfrac{s}{h r_\mathrm{m}} & 0 \le s < 2\pi R_\mathrm{ext} \\ N(s) = N_d F_\tau\,; & p_\mathrm{m}(s) = \dfrac{N_d F_\tau}{h r_\mathrm{m}} & 2\pi R_\mathrm{ext} \le s \le L - 2\pi R_\mathrm{ext} \\ N(s) = \dfrac{N_d F_\tau}{2\pi R_\mathrm{ext}} (L-s)\,; & p_\mathrm{m}(s) = \dfrac{N_d F_\tau}{2\pi R_\mathrm{ext}} \dfrac{L-s}{h r_\mathrm{m}} & L - 2\pi R_\mathrm{ext} \le s \le L \end{cases} . \tag{S.4}$$

In these expressions, R_ext indicates the radius of the (equal) external rungs of the helix and it is considered constant therein. Following the above assumptions and enforcing an axisymmetry approximation, it is possible to change the parametrization from the arc length s to the tubule axial coordinate $z = s\, h/(2\pi R) - H/2$ and radius r_m. Eventually, we obtain the z-dependence shown in the red graph of Figure 1 and reading

$$p_\mathrm{m}(r_\mathrm{m}, z) = \begin{cases} \dfrac{N_d F_\tau}{h\, r_\mathrm{m}} \dfrac{\tilde{h}}{h} & 0 \le |z| < H/2 - \tilde{h} \\ \dfrac{N_d F_\tau}{h\, r_\mathrm{m}} \dfrac{H/2 - |z|}{h} & H/2 - \tilde{h} \le |z| \le H/2 \\ 0 & \text{otherwise} \end{cases} , \tag{S.5}$$

where $\tilde{h} = \min(h, H-h)$ takes into account the case of short dynamins with $H < 2h$. As reasonable, this expression is valid for polymers that present at least one full turn, *i.e.* $H > h$.

Since the dynamin helix cannot constrict down to a null internal lumen of the PH domains due to its molecular structure [5, 13], we smoothly fade the pressure to zero between $r_\mathrm{m} \approx 3\,nm$ and $r_\mathrm{m} = 0$, as evidenced in the blue graph of Figure 1c. The specific choice of the decaying function is not relevant as long as it is smooth and does not affect the pressure profile for $r_\mathrm{m} > 3\,nm$. The modified mesoscopic pressure acting on the membrane (therefore oppositely oriented with respect to what shown in Figure S.1b) and entering the mathematical model in Equation 10 of the main text is ultimately expressed in the cylindrical coordinates r and z as

$$p(r,z) = \left(\tanh\frac{r}{\sqrt{3}}\right)^4 p_\mathrm{m}(r,z)\,. \tag{S.6}$$

The dynamin is supposed to depolymerize and stop its constricting action whenever the tubule ruptures [19] and we implement this feature by annihilating the pressure field as soon as the value $\phi(r=0,z) = -\tanh(3/\sqrt{2})$ is reached anywhere on the axis, *i.e.* when even the outermost surface of the bilayer is severed. Noticeably, in our model, no depolymerization energy is deposited on the lipid membrane, in accordance with recent findings [10, 20].

Lumen conductance

Among the available experimental techniques, measuring the conductance of the inner lumen of a tubule is a well-known, convenient, and quite precise (in terms of geometry and time resolution) mean to follow real-time constriction of lipid tubules [19, 21, 22]. In order to estimate the mobility of the evolution dynamics adopted in our numerical

approach and, additionally, propose an explanation for dynamin-driven constriction data, we compute and measure the lumen conductance in our simulations.

There exist different levels of accuracy in computing the conductance of a channel. In particular, when the cross-sectional dimensions of the system are in the order of a few nanometers, the accumulation of additional charge carriers in the Debye layer near the membrane walls influence the electric resistance of the lumen. Following the indications of [21–23], we compute the Debye corrected lumen conductance as

$$G = \frac{\pi e^2 D 2 C_b}{k_B T}\frac{r^2}{L}\sqrt{1+\frac{\sigma_c^2}{e^2 C_b^2 r^2}}\,. \tag{S.7}$$

Here, $D = 2\times 10^{-9}\,m^2 s^{-1}$ is the ion diffusivity, e the elementary charge, $C_b = 0.15\,M/L$ the bulk charges concentration, $\sigma_c = 0.5\,e\,nm^{-2}$ the charge density on the inner monolayer, r the radius of the inner lumen, and L the length of the tubule. The first ration in the conductance expression is usually called bulk specific resistivity, $\rho_b = \frac{k_B T}{e^2 D 2 C_b} \approx 66.7\,Ohm\,cm$. The reported values are adopted from the indications of [19, 21, 22]. This expression for the Debye-corrected conductance was proved to be effective for lumen radii down to $\sim 2\,nm$ in the case of mean-field approaches with charged capillary walls [23, 24]. Eventually, the normalized conductance of the numerical tubule lumen is computed as

$$G_n = \frac{L}{r_0\sqrt{r_0^2+\frac{\sigma_c^2}{e^2C_b^2}}}\left[\int_{-L/2}^{L/2}\left(r\sqrt{r^2+\frac{\sigma_c^2}{e^2C_b^2}}\right)^{-1}dz\right]^{-1}, \tag{S.8}$$

with r_0 the initial lumen radius.

Determining the mobility and reference time of the system

Though most of the results discussed in this work pertain to time-independent observables of the system, such as critical or equilibrium quantities, we are interested in assessing the physical meaning of the dissipative dynamics imposed to the order parameter. Therefore, we both validate the consistency of Equation 14 with real-time experimental observations as well as estimate the mobility of the system, M_{pf}. The selected benchmark is the time-resolved normalized conductance of the tubule lumen during constriction induced by osmotic pressure, provided by Bashkirov et al. in [19]. We reproduce the same setting by imposing $\gamma = 5\times 10^{-4}\,Nm^{-1}$ (a value taking into account the electric potential effects on surface tension [22]), $k_b = 16\,k_B T$, and $R_{\mathrm{in}} \approx 8\,nm$ thereof. The tubule length is $L = 1.4\,\mu m$ and the external osmotic pressure is computed as

$$p(r,z) = -N_{\mathrm{A}} k_B T\left[\left(\frac{(R_{\mathrm{in}}-l_{me}/2)^2}{(r-l_{me}/2)^2}-1\right)c_{\mathrm{in}}^0+\Delta c^0\right], \tag{S.9}$$

with N_{A} the Avogadro constant and l_{me} the membrane thickness. The initial osmotic concentration inside the tubule is c_{in}^0 and its difference with the external aqueous phase osmolarity is Δc^0 at the beginning. As shown in [19], the pressure exerted on the lipid bilayer is proportional to the osmotic concentration difference but, as pointed out in [25], the inner environment becomes more concentrated the more the tubule is constricted, thereby providing the dependence on r in Equation S.9. By imposing osmotic concentrations analogous to those in [19], *i.e.* $c_{\mathrm{in}}^0 = 0.22\,Osm$ and $\Delta c^0 = -0.38\,Osm$, we evolve the system and measure the Debye-corrected and normalized conductance of the lumen. Figure S.2 shows the comparison of the numerical and experimental conductances, G_n^{num} (blue dots) and G_n^{ex} (black shaded curve), respectively. From these, we are able to estimate the reference time of the numerical solution. In particular, we first fit the two curves with exponentially decaying functions with the same steady-state conductances and then match their decay times. Ultimately, we get the reference time $\tau_R = 0.005\,s$ for the numerical results. The non-dimensional form of Equation 14 provides a relationship between τ_R and the phase field mobility, resulting in $M_{\mathrm{pf}} = 4.04\,nm^3/(s\,k_B T)$. As will be demonstrated later on, this phase field mobility is, in general, linked to the sharp one, M_{sharp}, by the specific choice of ϵ.

Estimating the analytical fission time

Under the hypothesis of perfectly cylindrical tubule and related dynamics, with R being the radius of its mid-surface Γ, it is possible to explicitly link the order parameter to the geometry of the membrane. In particular, defining n the

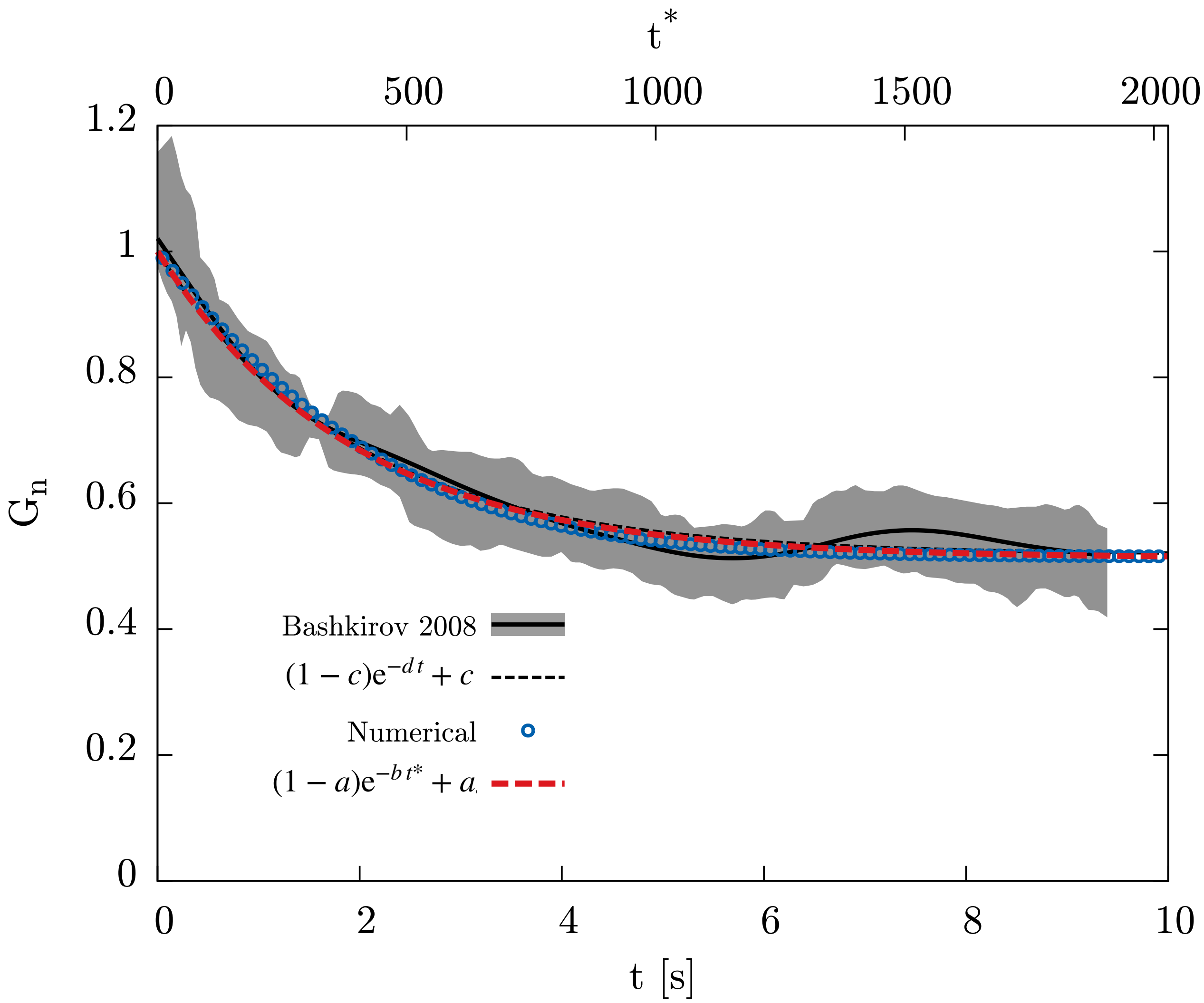

FIG. S.2. Reference time estimation through osmotic pressure constriction. The Debye-corrected and normalized conductance of the tubule internal lumen is shown during the time evolution of a tubule constricted by a hypertonic solution. Numerical (blue dots, with numerical time $t^\star$ on top axis) and experimental [19] (black shaded curve, with actual time on the bottom axis) results are shown. The same mesoscopic experimental conditions are reproduced in the simulation. Specifically, $\gamma = 5 \times 10^{-4}\, Nm^{-1}$, $k_b = 16\, k_B T$, and $R_{\rm in} \approx 8\, nm$ thereof. The osmotic pressure is computed as indicated by Equation S.9 in the text, with $c^0_{\rm in} = 0.22\, Osm$ and $\Delta c^0 = -0.38\, Osm$, the latter value granting the same steady state conductance. Exponentially decaying functions are used to fit the data (dashed black line for experimental and dashed red for numerical) in order to retrieve the ratio of the decay times, $b/d = \tau_R = 0.005\, s$. For the sake of completeness, the other fitting parameters are $a \approx b \approx 0.51$.

signed distance from Γ, we write

$$\phi(\boldsymbol{x}, t) = \phi(r, R(t)) = \phi(n(t)) \qquad \text{where } n(t) = r - R(t)\,. \tag{S.10}$$

If the tubule radius varies by a small amount, δR, the corresponding change expected on the order parameter is

$$\phi(r, R + \delta R) = \phi(r, R) + \left.\frac{\partial \phi}{\partial R}\right|_{r,R} \delta R\,, \tag{S.11}$$

$$\delta R \Rightarrow \delta \phi = -\frac{\partial \phi}{\partial n} \delta R\,. \tag{S.12}$$

On the other hand, we know that the free energy functional depending on the phase field, namely the Ginzburg-Landau free energy in Equation 3, converges to the sharp energy (Equation 1 augmented by a proper interaction contribution)

in the limit of vanishing ϵ. It is worth noticing that we are referring to a condition where the Canham-Helfrich theory is expected to hold since the hypothesis of small width-to-radius ratio is satisfied. As also supported by [26], not only the diffuse interface energy converges to the sharp one, but also its variation as long as it is determined by a suitable displacement (*i.e.* following a gradient flow). Defining $R_{\text{in}} = \sqrt{k_b/(2\gamma)}$ and given a certain δR, then

$$\delta F[\phi] = \int_\Omega \frac{\delta F}{\delta \phi} \delta\phi \, dV \overset{\epsilon/R_{\text{in}}\to 0}{\longrightarrow} \delta \mathcal{H}_{\text{sharp}}(R) = \frac{d\mathcal{H}_{\text{sharp}}(R)}{dR}\delta R \,, \tag{S.13}$$

where the sharp free energy function, $\mathcal{H}_{\text{sharp}}(R)$, consists in the Canham-Helfrich Hamiltonian augmented with an interaction term and only accounting for perfectly cylindrical configurations. In particular, the interaction is expressed by an inward pressure field, $p(R)$, depending on the sole radius, R, of the cylindrical membrane. It is then possible to write the sharp energy as

$$\mathcal{H}_{\text{sharp}}(R) = k_b \frac{\pi L}{R} + \gamma 2\pi R L + 2\pi L \int_{R_0}^{R} p(r) r \, dr \,, \tag{S.14}$$

upon exploiting $p(r)dV = p(r)2\pi r L dr$. Now, since

$$\frac{\partial \phi}{\partial t} = -M_{\text{pf}} \frac{\delta F[\phi]}{\delta \phi} \,, \tag{S.15}$$

we can compute

$$\int_\Omega \frac{\partial \phi}{\partial t} \delta\phi \, dV = -M_{\text{pf}} \int_\Omega \frac{\delta F[\phi]}{\delta \phi} \delta \phi \, dV \,, \tag{S.16}$$

which becomes

$$2\pi L \int_0^\infty r \left(\frac{\partial \phi}{\partial n}\right)^2 \frac{dR}{dt} \delta R dr = -M_{\text{pf}} \frac{d\mathcal{H}_{\text{sharp}}(R)}{dR} \delta R \,. \tag{S.17}$$

Noticing that $3\epsilon/(2\sqrt{2}) \left(\frac{\partial \phi}{\partial n}\right)^2 \overset{\mathcal{W}}{\longrightarrow} \delta(n)$ for $\epsilon/R_{\text{in}} \to 0$, we obtain

$$2\pi L \frac{2\sqrt{2}}{3\epsilon} R \frac{dR}{dt} \delta R = -M_{\text{pf}} \frac{d\mathcal{H}_{\text{sharp}}(R)}{dR} \delta R \tag{S.18}$$

and

$$\frac{dR}{dt} = -\frac{3\epsilon M_{\text{pf}}}{4\pi\sqrt{2} L R} \frac{d\mathcal{H}_{\text{sharp}}(R)}{dR} \,. \tag{S.19}$$

Ultimately, we retrieve the evolution equation for the tubule radius corresponding to the solution of the phase field dynamics in Equation S.15, reading

$$\frac{dR}{dt} = -\frac{3\epsilon}{4\sqrt{2}} M_{\text{pf}} \left(-\frac{k_b}{R^3} + \frac{2\gamma}{R} + 2p(R)\right) \,. \tag{S.20}$$

Equation S.20 can also be straightforwardly obtained from the Allen-Cahn dynamics based on the general sharp energy, $\mathcal{H}_{\text{sharp}}[\boldsymbol{u}]$, and reads

$$\frac{\partial \boldsymbol{u}}{\partial t} = -M_{\text{sharp}} \frac{\delta \mathcal{H}_{\text{sharp}}[\boldsymbol{u}]}{\delta \boldsymbol{u}} \,, \tag{S.21}$$

where

$$\mathcal{H}_{\text{sharp}}[\boldsymbol{u}] = \int_\Gamma f[\boldsymbol{u}] \, dS = \int_\Gamma \left[\frac{k_b}{2}(2M)^2 + \gamma\right] dS + \mathcal{H}_{\text{interaction}}[\boldsymbol{u}] \tag{S.22}$$

is the general Canham-Helfrich Hamiltonian augmented with the pressure contribution, $\mathcal{H}_{\text{interaction}}[\boldsymbol{u}]$. Exploiting $dS = j \, dS_0$, the functional derivative is

$$\frac{\delta \mathcal{H}_{\text{sharp}}[\boldsymbol{u}]}{\delta \boldsymbol{u}} = \frac{\delta f}{\delta \boldsymbol{u}} + \frac{f}{j} \frac{\delta j}{\delta \boldsymbol{u}} \,. \tag{S.23}$$

For the particular case of interest here, namely a cylindrical geometry where $\boldsymbol{u} = (R\cos\theta, R\sin\theta, z)$ and $\mathcal{H}_{\text{interaction}}[\boldsymbol{u}] = \int_\Gamma dS j^{-1} \int_{R_0}^{R} dr\, p(r) j(r) = 2\pi L \int_{R_0}^{R} dr\, p(r) r$, Equation S.23 is

$$\frac{\delta \mathcal{H}_{\text{sharp}}[\boldsymbol{u}]}{\delta \boldsymbol{u}} = \left[-\frac{k_b}{2R^3} + \frac{\gamma}{R} + p(R) \right] \boldsymbol{n}_\Gamma \,, \tag{S.24}$$

with $\boldsymbol{n}_\Gamma$, now, the outward pointing surface normal. Noticeably, recalling Equation S.14 and knowing that $R = \boldsymbol{u} \cdot \boldsymbol{n}_\Gamma$, Equations S.21 and S.24 provide the evolution equation for the cylinder radius

$$\frac{dR}{dt} = -\frac{M_{\text{sharp}}}{2\pi L R} \frac{d\mathcal{H}_{\text{sharp}}(R)}{dR} \,. \tag{S.25}$$

By comparison with Equation S.19, the diffuse and sharp mobilities are related by

$$M_{\text{sharp}} = M_{\text{pf}} \frac{3\epsilon}{2\sqrt{2}} = 3.57 \, \frac{nm^4}{s \; k_B T} \tag{S.26}$$

where $M_{\text{pf}} = 4.04\, nm^3/(s\, k_B T)$ (see previous sections) and $\epsilon = 5/6\, nm$ (see main text). Equation S.26 highlights the role of ϵ, namely the diffuse interface thickness, in relating the sharp and diffuse mobilities of the system.

Defining $\rho = R/R_{\text{in}}$, Equation S.20 reads

$$\frac{d\rho}{dt} = -M_{\text{sharp}} \frac{\gamma}{R_{\text{in}}^2} \frac{\rho^2 - 1 + p(R) R_{\text{in}} \gamma^{-1} \rho^3}{\rho^3} \,, \tag{S.27}$$

which, in the case of free, unforced tubule ($p(R) \equiv 0$), is solved by

$$\rho^2 + \log(|\rho^2 - 1|) = -2M_{\text{sharp}} \frac{\gamma}{R_{\text{in}}^2} t + \text{const} \,. \tag{S.28}$$

For an unforced tubule with $\rho \sim 1$ and $\rho \neq 1$, the solution is approximated by

$$\rho(t) \simeq 1 + (\rho(t=0) - 1) e^{-\frac{2\gamma M_{\text{sharp}}}{R_{\text{in}}^2} t} \,, \tag{S.29}$$

where the free relaxation time is

$$\tau_{\text{free}} = \frac{R_{\text{in}}^2}{2\gamma M_{\text{sharp}}} \,. \tag{S.30}$$

This same approach might be exploited in order to get an estimate of the fission time in the presence of a dynamin covering the entire tubule. In this scenario, the pressure is $p(R) = \frac{N_d F_\tau}{hR}$, and the solution gets

$$\rho^2 + \frac{\gamma}{\bar{\gamma}} \log(|\rho^2 - \frac{\gamma}{\bar{\gamma}}|) = -M_{\text{sharp}} \frac{2\gamma}{R_{\text{in}}^2} \frac{\bar{\gamma}}{\gamma} t + \text{const} \,, \tag{S.31}$$

where $\bar{\gamma} = \gamma + N_d F_\tau / h \gg \gamma$. Under the hypotheses of $\rho \sim 1$ and $\rho(t=0) = 1$, the approximate solution is

$$\rho(t) \simeq \sqrt{1 - \frac{2M_{\text{sharp}}}{R_{\text{in}}^2} \left(\frac{\bar{\gamma}^2}{\gamma + \bar{\gamma}} \right) t} \,. \tag{S.32}$$

Though it is valid only in the assumption of $\rho \sim 1$ and fully-covering coat, Equation S.32 approximates well enough the dynamics of the central and neck radii obtained from the complete numerical simulations for long dynamins ($H > H^\star$). Moreover, Equation S.32 provides the time for reaching $\rho = 0$, namely the characteristic fission time, which is expressed as

$$t_{\text{f}}^{\text{ch}} = \frac{R_{\text{in}}^2}{2M_{\text{sharp}}} \left(\frac{\gamma + \bar{\gamma}}{\bar{\gamma}^2} \right) \,. \tag{S.33}$$

Despite the good accordance in the constriction phase, supported by the results in Figure S.3, once the cylindrical structure approaches the normalized equilibrium radius, $\rho_{\text{eq}} = R_{\text{c}}/R_{\text{in}} = \sqrt{\gamma/\bar{\gamma}}$ shown as a plateau of the analytical solution in Figure S.3, fission proceeds through the sole necks located at the edges of the coat. Indeed, the equilibrium

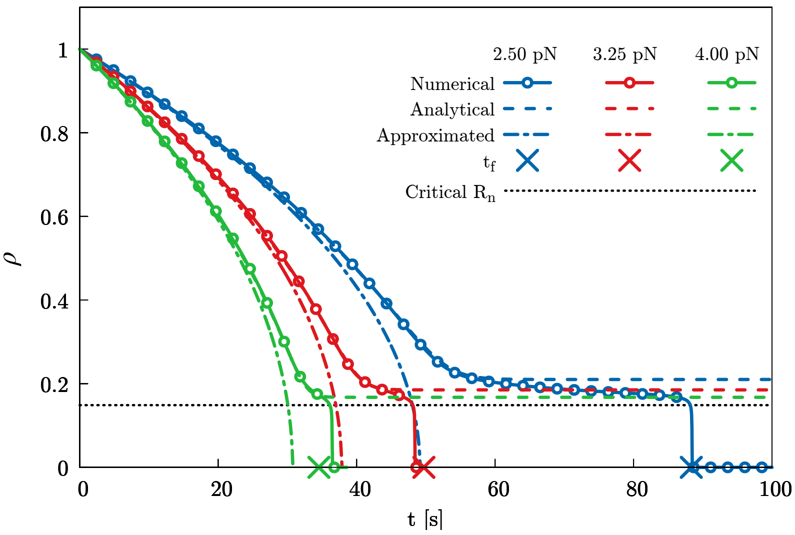

FIG. S.3. Fission time estimation for $H = 200\,nm$. The normalized (neck) radius evolution, $\rho(t)$, obtained from complete numerical simulations – lines with circles – is compared to the analytical solution obtained from Equation S.31 – dashed lines – and to the approximate solution in Equation S.32 – dash-dotted lines – for dynamins with $F_\tau = 2.5\,pN$, in blue, $F_\tau = 3.25\,pN$, in red, and $F_\tau = 4\,pN$, in green. The critical neck radius, $R_{\rm n} \simeq 2.5\,nm$ from Figure 2A of the main text, is shown for reference after normalization – black dotted line – and the corrected fission times, $t_{\rm f}$ in Equation S.35, are depicted by the cross symbols.

radius of this sharp cylindrical model, $R_{\rm c}$, is close to the critical value of the neck radius, $R_{\rm n} \simeq 2.5\,nm$, for the dynamin intensities selected here and additional effects start playing a role, leading to a mismatch between the numerical fission time and $t_{\rm f}^{\rm ch}$ that decreases with more intense constriction forces. Therefore, we introduce a fitted correction,

$$\alpha(F_\tau) = 1 + {\rm e}^{(2.318pN - F_\tau)/0.805pN}\,, \tag{S.34}$$

which modifies the expected fission time as

$$t_{\rm f} = \alpha(F_\tau)\frac{R_{\rm in}^2}{2M_{\rm sharp}}\left(\frac{\gamma + \bar{\gamma}}{\bar{\gamma}^2}\right)\,. \tag{S.35}$$

On the other hand, short dynamins ($H \leq H^\star$) do not present at all the cylindrical constriction and the above estimates fail. Still, with the aim of providing a convenient formula valid for all values of H and F_τ, we fit the fission times of numerical simulations and obtain the more general correction coefficient

$$\alpha(H, R_{\rm in}, F_\tau) = \begin{cases} 1 + 0.137\left(\dfrac{\sqrt{2}R_{\rm in}}{H - (11.871\,nm)}\right)^2 & 12\,nm \leq H < H^\star(R_{\rm in}) \\ 1 + {\rm e}^{(2.318pN - F_\tau)/0.805pN} & H \geq H^\star(R_{\rm in}) \end{cases}\,, \tag{S.36}$$

with $H^\star(R_{\rm in}) = (25.6\,nm) + 0.77R_{\rm in}$ as found in main text. The rationale behind the fitting function for short dynamins ($H < H^\star$) comes from the fact that edge effects decay as $\sim \sqrt{2}R_{\rm in}$ (see above sections for details). The accuracy of the fitted corrections is shown in Figure S.4 against the available numerical fission times for different values of the mesoscopic parameters. It is worth noticing that the correction is fairly small when dealing with intense constriction activity, *i.e.* $F_\tau \sim 4\,pN$, and long dynamins.

---

[1] K. Faelber, Y. Posor, S. Gao, M. Held, Y. Roske, D. Schulze, V. Haucke, F. Noé, and O. Daumke, Crystal structure of nucleotide-free dynamin, Nature **477**, 556 (2011).

[2] M. G. Ford, S. Jenni, and J. Nunnari, The crystal structure of dynamin, Nature **477**, 561 (2011).

[3] T. F. Reubold, K. Faelber, N. Plattner, Y. Posor, K. Ketel, U. Curth, J. Schlegel, R. Anand, D. J. Manstein, F. Noé, V. Haucke, O. Daumke, and S. Eschenburg, Crystal structure of the dynamin tetramer, Nature **525**, 404 (2015).

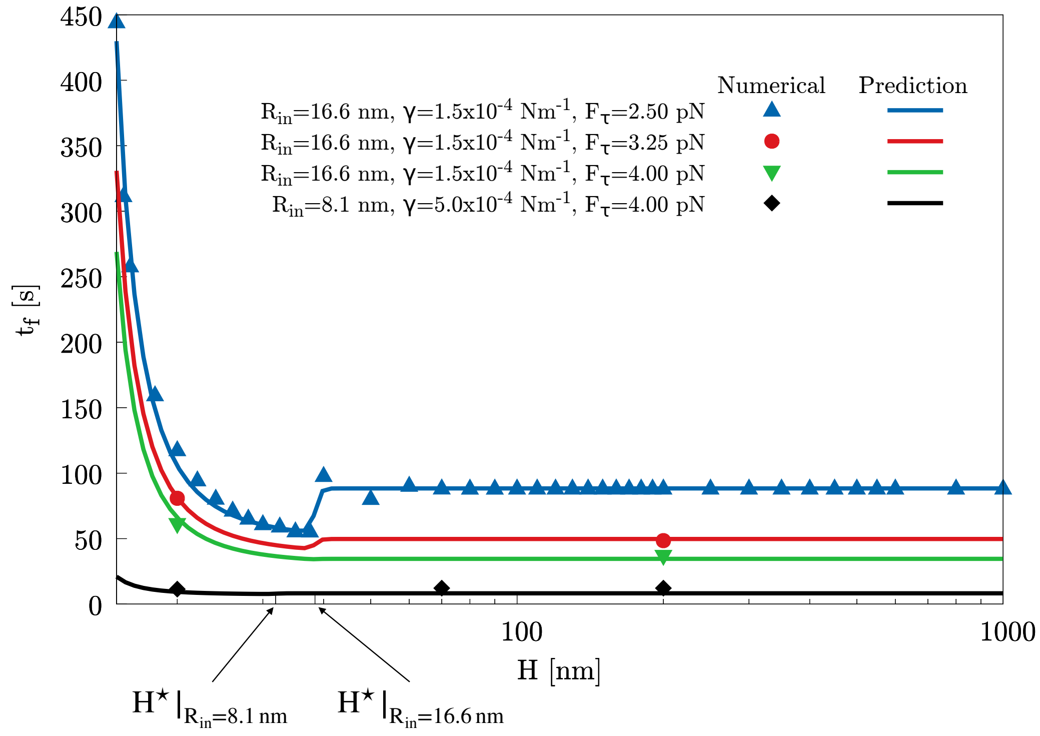

FIG. S.4. Fitted correction of the analytical fission time. Equation S.33 provides an analytical fission time for perfectly cylindrical systems. For actual cases, dynamins have a finite length, H, and membranes have a finite thickness, l_{me}, requiring a correction to be introduced therefor. The numerical fission times found in the diffuse interface simulation campaign are shown as symbols for different elastic parameters. Noticeably, the numerical fission time does not depend on H when $H > H^\star$ and the small, fitted correction only depends on F_τ. In the case of $H < H^\star$, the correction depends both on R_{in} and H. The solid lines show the fission time prediction modified by such corrections, see Equation S.36

[4] R. Ramachandran and S. L. Schmid, The dynamin superfamily, Current Biology **28**, R411 (2018).
[5] B. Antonny, C. Burd, P. De Camilli, E. Chen, O. Daumke, K. Faelber, M. Ford, V. A. Frolov, A. Frost, J. E. Hinshaw, T. Kirchhausen, M. M. Kozlov, M. Lenz, H. H. Low, H. McMahon, C. Merrifield, T. D. Pollard, P. J. Robinson, A. Roux, and S. Schmid, Membrane fission by dynamin: What we know and what we need to know, The EMBO Journal **35**, 2270 (2016).
[6] O. M. Ganichkin, R. Vancraenenbroeck, G. Rosenblum, H. Hofmann, A. S. Mikhailov, O. Daumke, and J. K. Noel, Quantification and demonstration of the collective constriction-by-ratchet mechanism in the dynamin molecular motor, Proceedings of the National Academy of Sciences **118**, 10.1073/pnas.2101144118 (2021).
[7] L. Kong, K. A. Sochacki, H. Wang, S. Fang, B. Canagarajah, A. D. Kehr, W. J. Rice, M.-P. Strub, J. W. Taraska, and J. E. Hinshaw, Cryo-EM of the dynamin polymer assembled on lipid membrane, Nature **560**, 258 (2018).
[8] A. C. Sundborger and J. E. Hinshaw, Dynamins and BAR Proteins-Safeguards against Cancer, Critical Reviews in Oncogenesis **20**, 475 (2015).
[9] X. Cheng, K. Chen, B. Dong, M. Yang, S. L. Filbrun, Y. Myoung, T. X. Huang, Y. Gu, G. Wang, and N. Fang, Dynamin-dependent vesicle twist at the final stage of clathrin-mediated endocytosis, Nature Cell Biology **23**, 859 (2021).
[10] A. Colom, L. Redondo-Morata, N. Chiaruttini, A. Roux, and S. Scheuring, Dynamic remodeling of the dynamin helix during membrane constriction, Proceedings of the National Academy of Sciences **114**, 5449 (2017).
[11] J. S. Chappie, J. A. Mears, S. Fang, M. Leonard, S. L. Schmid, R. A. Milligan, J. E. Hinshaw, and F. Dyda, A pseudoatomic model of the dynamin polymer identifies a hydrolysis-dependent powerstroke, Cell **147**, 209 (2011).
[12] J. K. Noel, M. Levi, M. Raghunathan, H. Lammert, R. L. Hayes, J. N. Onuchic, and P. C. Whitford, SMOG 2: A Versatile Software Package for Generating Structure-Based Models, PLoS Computational Biology **12**, 1 (2016).
[13] A. C. Sundborger, S. Fang, J. A. Heymann, P. Ray, J. S. Chappie, and J. E. Hinshaw, A Dynamin Mutant Defines a Superconstricted Prefission State, Cell Reports **8**, 734 (2014).
[14] J. Liu, F. J. D. Alvarez, D. K. Clare, J. K. Noel, and P. Zhang, CryoEM structure of the super-constricted two-start dynamin 1 filament, Nature Communications **12**, 1 (2021).

[15] Y. J. Chen, P. Zhang, E. H. Egelman, and J. E. Hinshaw, The stalk region of dynamin drives the constriction of dynamin tubes, Nature Structural and Molecular Biology **11**, 574 (2004).

[16] S. L. Schmid and V. A. Frolov, Dynamin: Functional design of a membrane fission catalyst, Annual Review of Cell and Developmental Biology **27**, 79 (2011).

[17] M. Fuhrmans and M. Müller, Coarse-grained simulation of dynamin-mediated fission, Soft Matter **11**, 1464 (2015).

[18] M. Pannuzzo, Z. A. McDargh, and M. Deserno, The role of scaffold reshaping and disassembly in dynamin driven membrane fission, eLife **7**, 1 (2018).

[19] P. V. Bashkirov, S. A. Akimov, A. I. Evseev, S. L. Schmid, J. Zimmerberg, and V. A. Frolov, GTPase Cycle of Dynamin Is Coupled to Membrane Squeeze and Release, Leading to Spontaneous Fission, Cell **135**, 1276 (2008).

[20] S. Morlot, V. Galli, M. Klein, N. Chiaruttini, J. Manzi, F. Humbert, L. Dinis, M. Lenz, G. Cappello, and A. Roux, Membrane Shape at the Edge of the Dynamin Helix Sets Location and Duration of the Fission Reaction, Cell **151**, 619 (2012).

[21] A. V. Shnyrova, P. V. Bashkirov, S. A. Akimov, T. J. Pucadyil, J. Zimmerberg, S. L. Schmid, and V. A. Frolov, Geometric Catalysis of Membrane Fission Driven by Flexible Dynamin Rings, Science **339**, 1433 (2013).

[22] P. V. Bashkirov, P. I. Kuzmin, K. Chekashkina, P. Arrasate, J. Vera Lillo, A. V. Shnyrova, and V. A. Frolov, Reconstitution and real-time quantification of membrane remodeling by single proteins and protein complexes, Nature Protocols **15**, 2443 (2020).

[23] I. Vlassiouk, S. Smirnov, and Z. Siwyt, Ionic selectivity of single nanochannels, Nano Letters **8**, 1978 (2008).

[24] D. N. Petsev and G. P. Lopez, Electrostatic potential and electroosmotic flow in a cylindrical capillary filled with symmetric electrolyte: Analytic solutions in thin double layer approximation, Journal of Colloid and Interface Science **294**, 492 (2006).

[25] S. U. Alam Shibly, C. Ghatak, M. A. Sayem Karal, M. Moniruzzaman, and M. Yamazaki, Experimental Estimation of Membrane Tension Induced by Osmotic Pressure, Biophysical Journal **111**, 2190 (2016).

[26] S. Serfaty, Gamma-convergence of gradient flows on Hilbert and metric spaces and applications, Discrete and Continuous Dynamical Systems **31**, 1427 (2011).
